# NUFIP and the HSP90/R2TP chaperone bind the SMN complex and facilitate assembly of U4-specific proteins

**DOI:** 10.1093/nar/gkv809

**Published:** 2015-10-10

**Authors:** Jonathan Bizarro, Maxime Dodré, Alexandra Huttin, Bruno Charpentier, Florence Schlotter, Christiane Branlant, Céline Verheggen, Séverine Massenet, Edouard Bertrand

**Affiliations:** 1 Equipe labélisée Ligue contre le Cancer, Institut de Génétique Moléculaire de Montpellier, IGMM-UMR 5535 du CNRS-Université de Montpellier, 1919, route de Mende, 34293 Montpellier Cedex 5, France; 2 Ingénierie Moléculaire et Physiopathologie Articulaire, UMR 7365 CNRS-Université de Lorraine, Biopôle de l'Université de Lorraine, avenue de la forêt de Haye, BP 184, 54505 Vandoeuvre-les-Nancy Cedex, France

## Abstract

The Sm proteins are loaded on snRNAs by the SMN complex, but how snRNP-specific proteins are assembled remains poorly characterized. U4 snRNP and box C/D snoRNPs have structural similarities. They both contain the 15.5K and proteins with NOP domains (PRP31 for U4, NOP56/58 for snoRNPs). Biogenesis of box C/D snoRNPs involves NUFIP and the HSP90/R2TP chaperone system and here, we explore the function of this machinery in U4 RNP assembly. We show that yeast Prp31 interacts with several components of the NUFIP/R2TP machinery, and that these interactions are separable from each other. In human cells, PRP31 mutants that fail to stably associate with U4 snRNA still interact with components of the NUFIP/R2TP system, indicating that these interactions precede binding of PRP31 to U4 snRNA. Knock-down of NUFIP leads to mislocalization of PRP31 and decreased association with U4. Moreover, NUFIP is associated with the SMN complex through direct interactions with Gemin3 and Gemin6. Altogether, our data suggest a model in which the NUFIP/R2TP system is connected with the SMN complex and facilitates assembly of U4 snRNP-specific proteins.

## INTRODUCTION

Splicing is an essential process that removes introns from pre-mRNAs. It is catalyzed by the spliceosome, a complex molecular machine that assembles on each intron to be spliced (1–3). Small nuclear RNAs (snRNAs) are essential components of the splicing machinery. They orchestrate assembly of the spliceosome and form a key part of its catalytic center. In particular, U6 is believed to be directly involved in catalysis, possibly by positioning key metal ions that stabilize leaving groups during the trans-esterification reactions (4). U6 is incorporated in the spliceosome as part of a tri-snRNP also containing the U4 and U5 snRNPs (1–3). U4 extensively base-pairs with U6 and its release from U6 is crucial for spliceosome activation. U4 thus functions as a U6 chaperone, likely preventing undesired activities of free U6 and delivering it to the spliceosome in a form compatible with the formation of an active catalytic core (5). Because the U4/U6-U5 tri-snRNP dissociates during splicing, it has to be reassembled following every splicing reaction.

With the exception of U6, snRNPs contain a heptameric Sm ring and a variable number of snRNP-specific proteins (1–3). Alteration of snRNP biogenesis can lead to diseases and has thus been extensively studied (6–8). Pol-II transcribed snRNAs contain an m^7^G cap and are exported to the cytoplasm as a complex with CBC, PHAX, ARS2 and the exportin CRM1 (9,10). They are then loaded on the SMN complex, a machinery that functions as a clamp-loader to assemble the Sm ring around snRNAs (11–14). The SMN complex is formed by SMN, Gemin2–8 and Unrip proteins. *In vitro*, Gemin2 is directly involved in the assembly of the Sm ring (12–14), and the other Gemins are thought to play auxiliary roles during the *in vivo* reaction (15–20). Once the Sm core has been assembled, the m^7^G cap is hyper-methylated into m_3_G (TMG) and the snRNPs are reimported into nuclei by a complex containing Snurportin and Importin β (21,22). There, snRNPs first go to Cajal bodies (CBs) for final maturation steps, which include nucleotide modifications catalyzed by scaRNAs and formation of the U4/U6-U5 tri-snRNP (23–26). Despite this knowledge, we still have a poor understanding of the assembly of snRNP-specific proteins.

Among the five snRNPs, U6 has a unique maturation pathway (for review, 27). The U6 snRNA is synthesized by pol III, acquires a γ-monomethyl cap, and stays in the nucleus where it binds SART3 and a preformed ring of Lsm (Like Sm) proteins to form the U6 snRNP. Then, the Lsm and SART3 proteins facilitate formation of the U4/U6 di-snRNP, before assembly of U5 to form the U4/U6-U5 tri-snRNP (26,28–30). U4 plays a key role in the formation of the tri-snRNP and in vitro, it can form a specific RNP with the 15.5K protein at its heart (31,32). The 15.5K recognizes a specific K-turn on U4 snRNA and allows recruitment of PRP31 (33–35). The ternary complex then recruits PRP3, PRP4 and CYPH, likely during formation of the U4/U6-U5 tri-snRNP (33). Interestingly, U4 snRNP has similarities with box C/D snoRNPs (34). Both RNPs contain the 15.5K, and PRP31 is structurally similar to NOP56 and NOP58, two core proteins of the box C/D snoRNPs. These three proteins possess an NOP and a coiled-coil domain. The NOP domain binds to preformed 15.5K:RNA complexes (36), while the coiled-coil domain is important for protein–protein interactions: between NOP56 and NOP58 in the case of C/D snoRNPs (37), and with the U5–102K (hPrp6) protein in the case of U4 (36,38).

Box C/D snoRNPs are assembled by the HSP90/R2TP system with the aid of two adaptors: NUFIP and ZNHIT3 (Rsa1 and Hit1 in yeast) (39–41). The R2TP complex functions as a co-chaperone for HSP90 and contains four proteins (39,42): RPAP3 (Tah1p in yeast), PIH1D1 (Pih1p in yeast), and the two essential AAA+ ATPases RuvBL1 and RuvBL2 (Rvb1/2p in yeast; see Table 1 for nomenclature). During assembly of C/D snoRNP, NUFIP directly binds 15.5K and is thought to bridge it to the R2TP complex through its interaction with PIH1D1 (39). Interestingly, NUFIP was reported to also interact with PRP31, and NUFIP, R2TP proteins, and HSP90 are all associated with U4 snRNA in HeLa cells (39). Here, we explore in more details the interactions between U4 snRNP and the C/D box snoRNP assembly factors. We show that NUFIP participates in the assembly of PRP31 on U4 snRNA and that NUFIP and PRP31 are associated in cells with the SMN complex, suggesting a relationship between the assembly of the Sm-core and snRNP-specific proteins.

**Table 1.** Correspondence between the yeast and human proteins implicated in this study

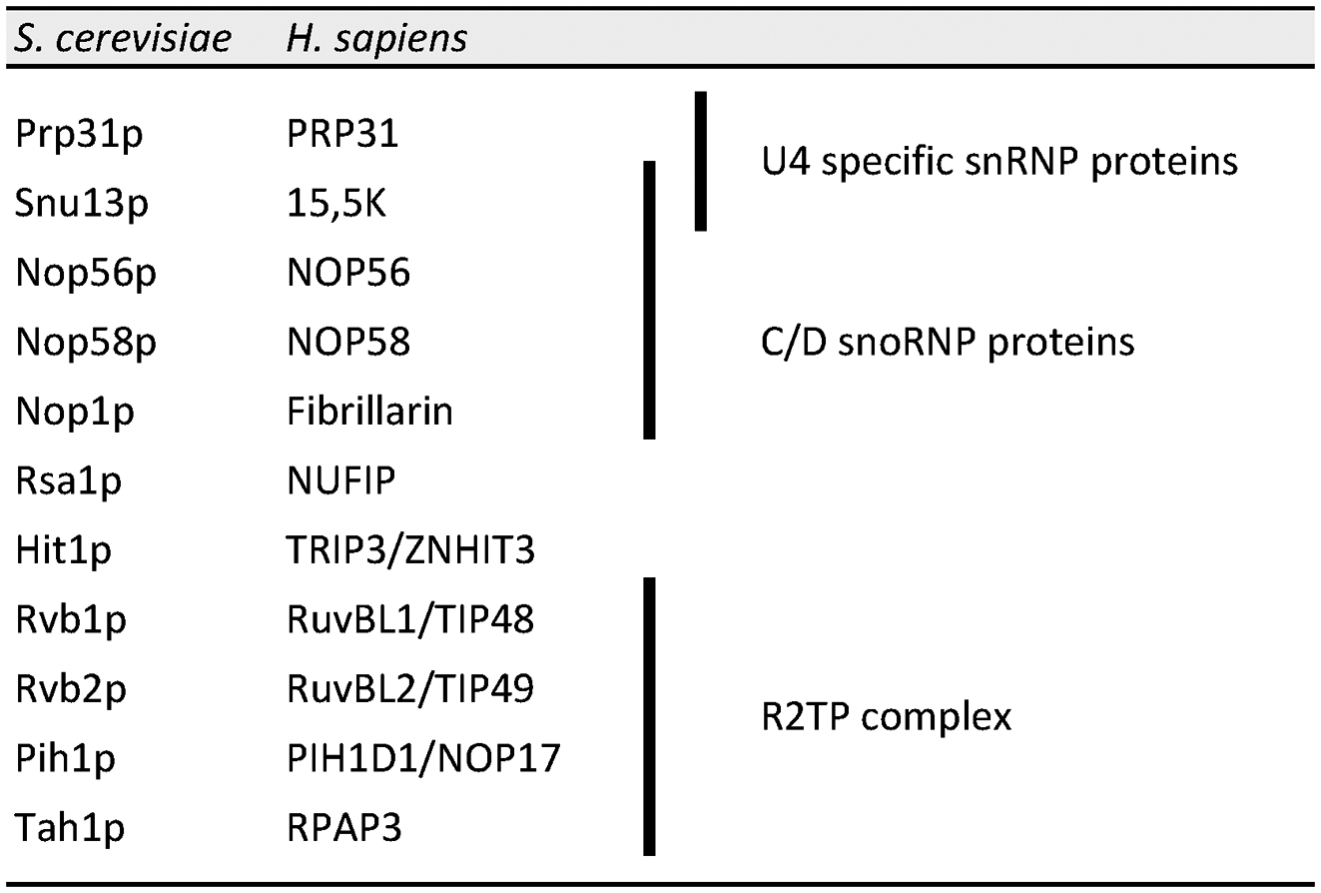		

## MATERIALS AND METHODS

### Cell culture, siRNAs and DNA manipulations

HeLa, U2OS, U2OS-LacO and 293T cells were maintained in Dulbecco's modified Eagle's medium supplemented with 10% of fetal bovine serum, penicillin/streptomycin (10 U/ml) and glutamin (2.9 mg/ml), in a humidified CO_2_ incubator at 37°C. Cells were transfected with the indicated plasmids or siRNAs for 48 h with JetPrime (Polyplus), following manufacturer recommendations. HeLa stable cell lines expressing GFP-PRP31 were created with the Flp-in system, as recommended by the manufacturer, using the plasmid pcDNA5-GFP-PRP31 (see below). SiRNA against NUFIP and control FFL siRNA had the following sequences respectively: 5′-GGAGCAGUAUUGACAACAAdTdT-3′ and 5′-UCGAAGUACUCAGCGUAAGdTdT-3′.

U4-MS2 was created from the U4C gene, by introducing by polymerase chain reaction (PCR) an MS2 stem-loop in the central stem-loop immediately upstream of the Sm site (corresponding to nucleotides 99–117 in Figure 3). GFP-NUFIP and Flag-SMN vectors were described previously (11,43). Two-hybrid plasmids, RFP-LacI-NLS-Gemin6, RFP-LacI-NLS-KpnA2, L30-GFP-NUFIP, L30-GFP-PRP31, L30-GFP-NOP58, L30-GFP-15.5K, L30-GFP-ZNHIT3, pcDNA5–3xFlag-PRP31, pcDNA5-GFP-PRP31, CMV-GST-PRP31, CMV-GST-15.5K, CMV-GST-NUFIP, pDEST17-Gemin3, pDEST17-Gemin4 and CMV-GST-ZNHIT3 plasmids were obtained using the Gateway technology (Invitrogen) with appropriate destination vectors (pACT2-Rf, pAS2ΔΔ-Rf, pL30-eGFP-Rf, pL30-RFP-LacI-NLS-Rf, pcDNA5–3xFlag-Rf, pcDNA5-GFP-Rf, pDEST15, pDEST17, maps available at http://www.igmm.cnrs.fr/spip.php?rubrique89&lang=en). Plasmids for expression of Gemin3, 4, 6 and 15.5K in *Escherichia coli* were obtained by PCR cloning of the corresponding ORFs into pnEA. The plasmid used to express GST-NUFIP in *E. coli* was described previously (43). Mutagenesis were performed with the QuickStrand mutagenesis kit (Stratagene), according to recommendations of the manufacturer. Detailed maps and sequences are available upon request.

### Yeast two hybrid assays

For Y2H assays, appropriate pACT2 and pAS2ΔΔ plasmids were introduced into haploid *S. cerevisiae* test strains (CG1945 and Y187, respectively), which were then crossed. Diploids were selected on –Leu –Trp media and then plated on test plates lacking amino acids Leu, Trp and His, and containing gradual amounts of 3-Amino-1,2,4-triazol (3-AT), which is a competitive inhibitor of the product of the *HIS3* gene. This was used to evaluate the strength of the interactions. Growth was assessed after three or four days of incubation at 30°C.

### *In vitro* binding experiments

GST-NUFIP was expressed in *E. coli* BL21(DE3) pRARE2 cells (Novagen) and purified by affinity chromatography on glutathione-Sepharose beads (GE healthcare). Gemin3, 4, 6 and 15.5K proteins were expressed in *E. coli* BL21(DE3) pRARE2 cells (Novagen) and total extracts were prepared in RSB 200 buffer (10 mM Tris-HCl pH 7.4, 2.5 mM MgCl_2_, 200 mM NaCl, 0,01% Igepal). RNase A (0.2 mg/ml) was added and the mixture was incubated for 15 min at 30°C prior to the binding experiments. Four μg of GST or GST-NUFIP proteins bound to glutathione-Sepharose beads were incubated with these extracts, for 1 h at 4°C. Beads were washed three times in RSB 200 buffer, eluted in Laemmli, and proteins were analyzed by western blotting using anti-Gemin3 (44), anti-Gemin4 (45), anti-Gemin6 (12307–2-AP, Proteintech) and anti-15.5 K (15802–1-AP, Proteintech) antibodies.

To analyze the interactions of *in vitro* translated proteins with the SMN complex, native SMN complexes were affinity purified using a HeLa Tet-Off cell line stably expressing Flag-tagged Gemin2 as described previously (46,47). SMN complexes were then incubated with *in vitro* translated [^35^S]methionine-labeled proteins in RSB 100 buffer (10 mM Tris-HCl pH 7.4, 2.5 mM MgCl_2_, 100 mM NaCl, 0,01% Igepal) for 2 h at 4°C. Following three washes with RSB 100 buffer, bound proteins were analyzed by SDS-PAGE followed by autoradiography.

### Immuno-precipitation experiments

Cells were extracted in HNTG buffer (20 mM HEPES pH 7.9, 150 mM NaCl, 1% Triton, 10% glycerol, 1 mM MgCl_2_, 1 mM EGTA, and protease inhibitors) for 30 min at 4°C. When indicated, RNase A was added at 60 μg/ml. Cellular debris were removed by centrifugation (10 min at 9000 g and at 4°C). Extracts were put on coated beads for 2 h at 4°C (GFP-Trap from Chromotek for GFP; Gluthathione sepharose 4B beads from GE Healthcare for GST). Beads were washed four times in HNTG. For protein analyses, pelleted materials were resuspended in Laemmli buffer and analyzed by western blotting using the following antibodies: mouse M2 monoclonal anti-Flag (Sigma; 1/5000), anti-15.5K (gift of J. Cavaillé, 1/1000), anti-ZNHIT3 (Abcam, 1/5000), anti-NUFIP (PTG Lab, 1/1000), anti-RuvBL1 (Proteintech, 1/2000), anti-RuvBL2 (Proteintech, 1/1000), anti-GFP (Roche, 1/5000), anti-Tubulin (University of Iowa, 1/500), anti-GAPDH (Abcam, 1/5000), anti-SMN (BD, 1/500).

For analyzing immunoprecipitated RNAs, the pelleted affinity beads were homogenized in Trizol (InVitrogen), and RNAs were purified according to the manufacturer instructions. RNase protection assays were performed with RPAIII kit (Ambion) following the recommended procedure. The probe sequence covers U4-MS2 on the last 147 nucleotides of the RNA as indicated in Figure 3B. For the endogenous U4, the probe protects regions of 56 and 43 nucleotides.

### Transposon mutagenesis of Prp31p and interaction screen

MuA-mediated mutagenesis was performed with the MGS kit (FinnZyme), according to the manufacturer instructions. The pAS2-*PRP31* plasmid was used as a substrate for the *in vitro* transposition reaction. Clones with MuA integration were selected on kanamycin, 5000 clones were pooled, digested with NotI to excise MuA, religated and transformed into bacteria and then yeast two-hybrid strain (CG1945). 500 clones were individually picked (equivalent to one insertion every 4 amino-acids, taking into account that MuA integration can occur everywhere in the plasmid), and tested for interaction with Rsa1p after mating with the Y187 strain containing the pACT2-*RSA1* plasmid. Diploids were selected on -Leu-Trp media and interaction was tested by growing diploids on -Leu-Trp-His plates containing 1.5 mM 3AT. Plasmids were then isolated from clones defective for the interaction with Rsa1p, re-transformed in the original CG1945 strain and tested for interaction as above, by mating with Y187 strains containing pACT2-*RSA1*, pACT2-*HIT1*, pACT2-*RVB1*, pACT2-*RVB2*, pACT2-*PIH1* and pACT2-*ALIX* as control. Plasmids defective for at least one of these interactions were isolated and sequenced.

### Subcellular localization assay

The co-recruitment assay was done as previously described (48). *In situ* proximity ligation assay (PLA) was performed as recommended by the manufacturer (DuolinkII kit, Olink Bioscience AB). Briefly, HeLa cells grown on coverslips were fixed in PBS 1X/paraformaldehyde 4% and permeabilized with a PBS 1X/ Triton X100 0.1% solution. Primary antibodies were diluted in 1x antibody diluent and incubated for 1 h at room temperature. The negative controls used only one of each primary antibody. Cells were washed twice for 5 min in PBS 1X. The PLA probes (Rabbit-MINUS and Mouse-PLUS) were incubated in a pre-heated humidity chamber for 1 h at 37°C. Subsequent steps were performed using the detection reagents green according to DuolinkII kit protocol. The Duolink mounting medium was supplemented with 10 μM TO-PRO-3 final to counterstain for nuclei. Laser confocal microscopy was performed with a SP5-AOBS X Leica confocal microscope. Images from each channel were recorded separately and then merged. Images were processed and assembled with Photoshop CS5 (Adobe). The antibodies used in these experiments were as follows: anti-SMN (2B1), anti-Gemin2 (2E17), anti-Gemin3 (44), anti-Gemin4 (45), anti-Gemin6 (12307–2-AP, PTG Lab), anti-Gemin7 (6E2, Millipore), anti-Gemin8 (1F8) (20), anti-NUFIP (12515–1-AP, Proteintech) and anti-GAPDH (Abcam).

### Fluorescence microscopy and image acquisition and quantification

Cells were grown on coverslips, washed in PBS and fixed in PBS1X/paraformaldehyde 4% at room temperature for 20 min, followed by permeabilization either with PBS 1X/ Triton X100 0.1% for 5 min at RT for antibody labeling, or with ethanol 70%, overnight at 4°C for in situ hybridization, which was performed with Cy3 labeled oligonucleotides against U85 as previously described (49). Coverslips were mounted on glass slides in Vectashield and samples were observed using a Leica DMRA microscope. Images were acquired with a Coolsnap HQ2 camera. The camera and microscope were driven by the Metamorph (Universal Imaging) software. For quantification, signals in CBs and nucleoplasm were background substracted and divided by each other.

## RESULTS

### An insertional mutagenesis screen identifies a mutant of yeast Prp31p that specifically loses its interaction with Hit1p.

**Figure 1.**
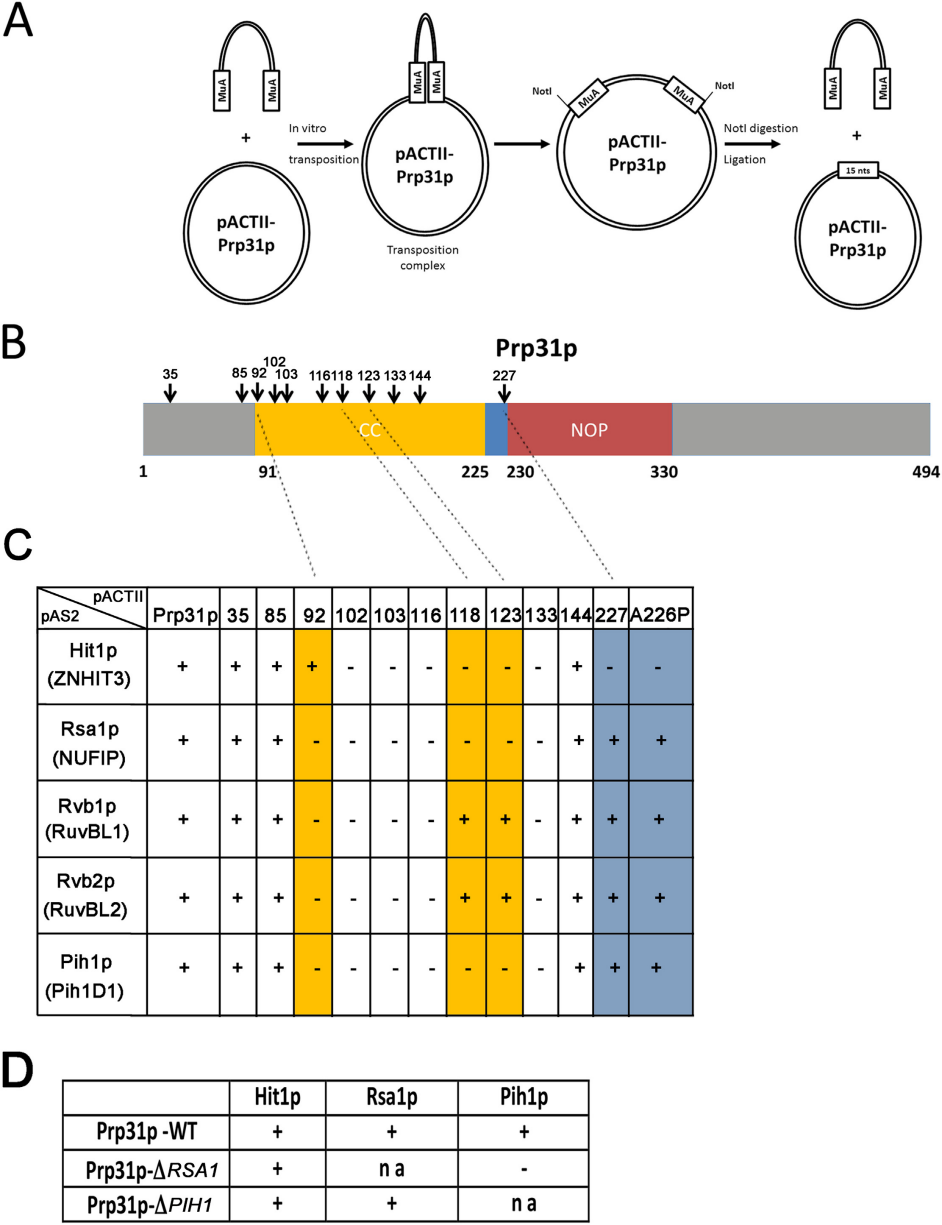
Identification and Y2H characterization of yeast Prp31p mutants. **(A)** Schematic representation of the insertional mutagenesis screen. Random insertion of MuA and excision by *Not*I digestion and religation leave a 5 nucleotide insertion at site of integration. **(B**) Schematic representation of Prp31p domains and location of the insertions that were identified in primary two-hybrid screen. **(C)** Summary of the two-hybrid validation screen. ‘+’ and ‘-’ indicate the presence or absence of interactions. The numbers of the pACTII plasmids indicate the amino-acid after which the 5 nucleotide insertion occurred in yPRP31. The proteins encoded by pAS2 plasmids are indicated, with in parenthesis the name of the human homologs. **(D)** Two-hybrid interactions in WT yeast strain or in yeast strains deleted for Rsa1 (Δ*RSA1*) or Pih1 (Δ*PIH1)*. The pACTII-yPRP31 plasmid was introduced in the indicated strain and tested against the indicated proteins. ‘na’: not applicable.

Using two-hybrid tests, we previously showed that yeast PRP31 (Prp31) interacts with a number of components of the yeast R2TP/NUFIP machinery (39). Indeed, positive interactions were found with Rsa1 (the yeast homolog of NUFIP), and with the R2TP components Pih1, Rvb1, Rvb2 (see Table 1 for correspondence with between the yeast and human nomenclature). We also found an additional interaction with Hit1, the yeast homolog of ZNHIT3 that was recently found to participate to C/D snoRNP biogenesis (see below; 40,41). To decipher the function of these interactions, we looked for mutants and performed a mutagenesis screen of yeast Prp31 using the MuA transposon (Figure 1A). MuA inserts randomly into DNA *in vitro*, and after excision of the transposon by *Not*I digestion, 15 nucleotides remain and create a 5-codon insertion. The Prp31 mutant library was transformed in yeast 2-hybrid strains, and 500 mutants (1 insertion every 4 amino-acids) were individually screened for interaction with Rsa1. Plasmids from mutants defective in binding were isolated, re-transformed and then tested against all interactants of Prp31: Rsa1, Pih1, Rvb1, Rvb2, and Hit1.

**Figure 2.**
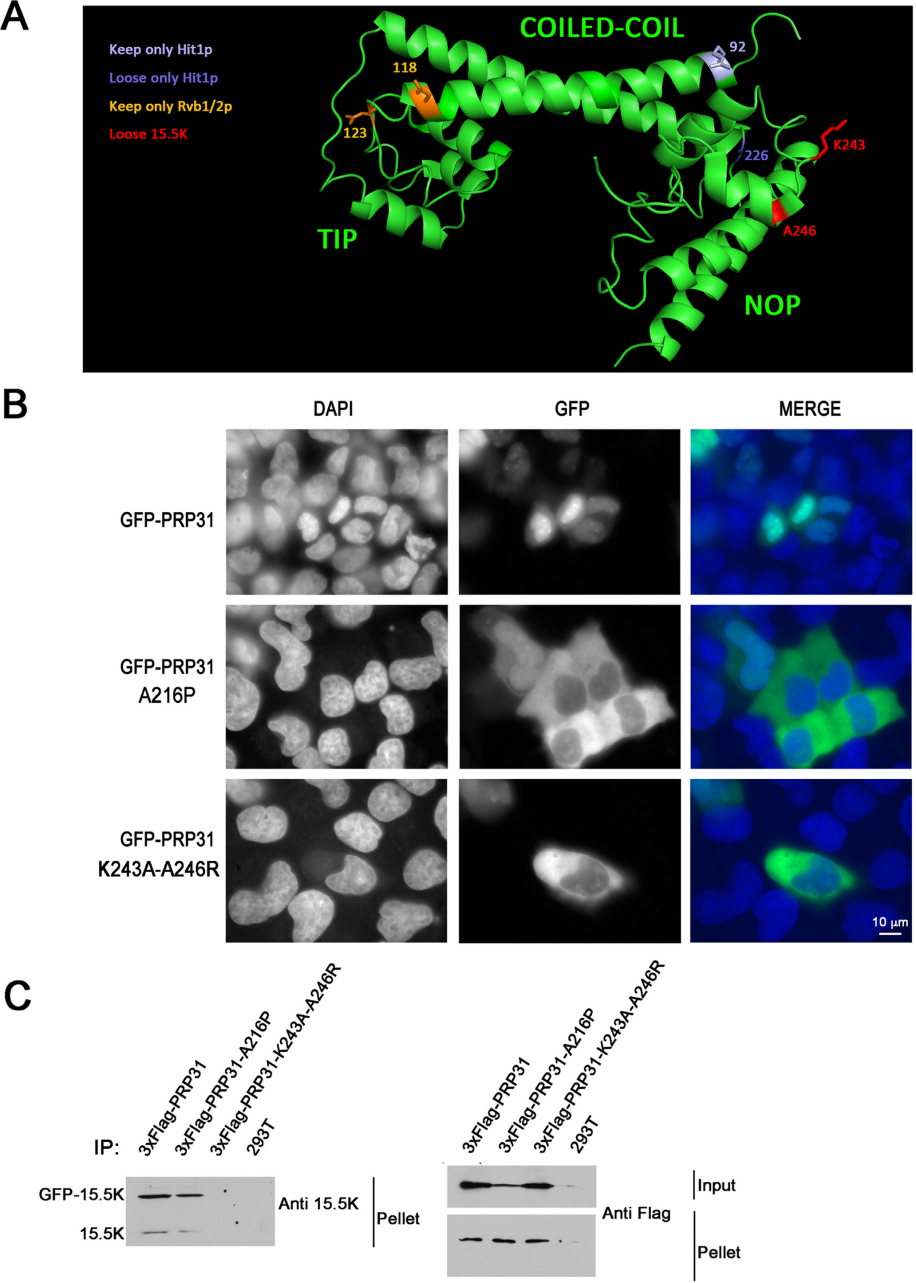
PRP31 A216P and PRP31 K243A-A246R are mostly cytoplasmic in human cells. **(A)** 3D Structure of human PRP31 with various mutations indicated. In yellow, amino-acids corresponding to MuA insertion sites leading to the loss of interaction with Pih1, Rsa1 and Hit1 (numbers correspond to the yeast protein). In blue, amino-acids corresponding to MuA insertion sites related to Hit1 (numbers correspond to the yeast protein). In red, amino acids mutated to prevent binding of 15.5K (numbers correspond to the human protein). **(B)** Micrographs showing the localization of wild-type and mutant PRP31 fused to GFP in U2OS cells. Scale bar is 10 μm. Blue: DAPI staining corresponding to nuclei; green: GFP-tagged PRP31 proteins. **(C)** PRP31 K243A-A246R does not interact with 15.5K in human cells. Western blotting of inputs and pellets of anti-Flag immuno-precipitates of 293T cells co-transfected with 3xFlag-PRP31 (wild-type and indicated mutants), GFP-15.5K or untransfected (293T). Western blots were probed with the indicated antibodies. Input: 5% of pellet.

The primary screen identified 11 mutants of Prp31 that no longer interacted with Rsa1, and 7 were confirmed to be defective in validation assays (Figure 1B and C). The corresponding insertions were located in the coiled-coil domain of Prp31p, confirming the central role of this domain. Four mutants lost interactions with all the proteins tested. Interestingly however, two mutants (a.a. 118 and 123) lost interactions with Rsa1, Pih1 and Hit1, but were still interacting with Rvb1 and Rvb2. These two mutations were located very near or within the globular tip domain at the end of the coiled-coil (Figures 1B and 2A). This indicated that the coiled-coil domain was required for interaction with Rvb1/2, and that the globular tip was required for interaction with Rsa1, Pih1 and Hit1. To further dissect the interaction network, we performed Y2H assays in strains deleted for Rsa1 or Pih1 (Figure 1D). We found that the interaction of Prp31 with Pih1 was dependent on Rsa1, while interaction with Hit1 was independent of both Rsa1 and Pih1. The two remaining mutants confirmed that Hit1 interacted with Prp31 independently from the other proteins. Indeed, insertion at the very beginning of the coiled-coil domain led to loss of all interactions except that of Hit1p (a.a. 92). Conversely, insertion at the junction between the coiled-coil and the NOP domain lost only the interaction with Hit1 (a.a. 227; see Figure 2A). The results of this screen thus indicated that the interactions of Prp31 with Rvb1/2, Hit1 or Rsa1 are separable from each other, suggesting that they occupy different binding surfaces.

The insertion mutant at amino acid 227 was especially intriguing because this is only one amino-acid away from a mutation of human PRP31 that occurs in retinitis pigmentosa (50), and which changes alanine 216 into a proline (corresponding to a.a 226 in yeast Prp31p). Retinitis pigmentosa is a group of inherited diseases characterized by the gradual degeneration of retina cells that lead to night blindness and visual field loss (for review, 51). Our results prompted us to test the A226P mutant of yeast Prp31p in our Y2H assays (the position homologous to A216P in the human protein). In agreement with the screen, we found that this mutant only lost the interaction with Hit1p (Figure 1C). The occurrence of this mutation in a human disease pointed toward a functionally important role for the interaction between Hit1p and Prp31p.

### PRP31 A216P and mutants defective for association with 15.5K accumulate in the cytoplasm of human cells

Because of the link between the PRP31 A216P mutation and retinitis pigmentosa, we turned to human cells to study U4 biogenesis in more details. First, we fused wild-type and mutants PRP31 to GFP and analyzed their localization in U2OS cells (Figure 2B). While wild-type PRP31 accumulated in the nucleus, the mutant protein A216P was mostly located in the cytoplasm, in agreement with previous studies (Figure 2B; 52,53). This mutant associated in cells with 15.5K (Figure 2C) even though its ability to be incorporated in U4/U6 snRNPs was shown to be dramatically reduced (53). Because the A216P mutation is expected to change the orientation of the NOP domain with respect to the coiled-coil, we tested the effect of a more subtle mutation. The NOP domain of PRP31 directly interacts with the 15.5K protein via amino-acids located at the surface of the protein, which do not participate to its overall folding (Figure 2A; 36). We mutated two amino-acids of PRP31 directly involved in this interaction, K243 and A246. In co-IP experiment, the mutant protein was indeed unable to interact with the 15.5K (Figure 2C). Remarkably, this mutant PRP31 protein also accumulated preferentially in the cytoplasm, with only low nuclear levels (Figure 2B). Since PRP31 bears an NLS and since the A216P mutants is imported at normal rates (54), these data suggest that the PRP31 proteins unable to stably incorporate into snRNPs are either re-exported to the cytoplasm, or are rapidly degraded following their nuclear import.

### Wild-type and mutant PRP31 weakly associate with U4 RNA in absence of 15.5K

**Figure 3.**
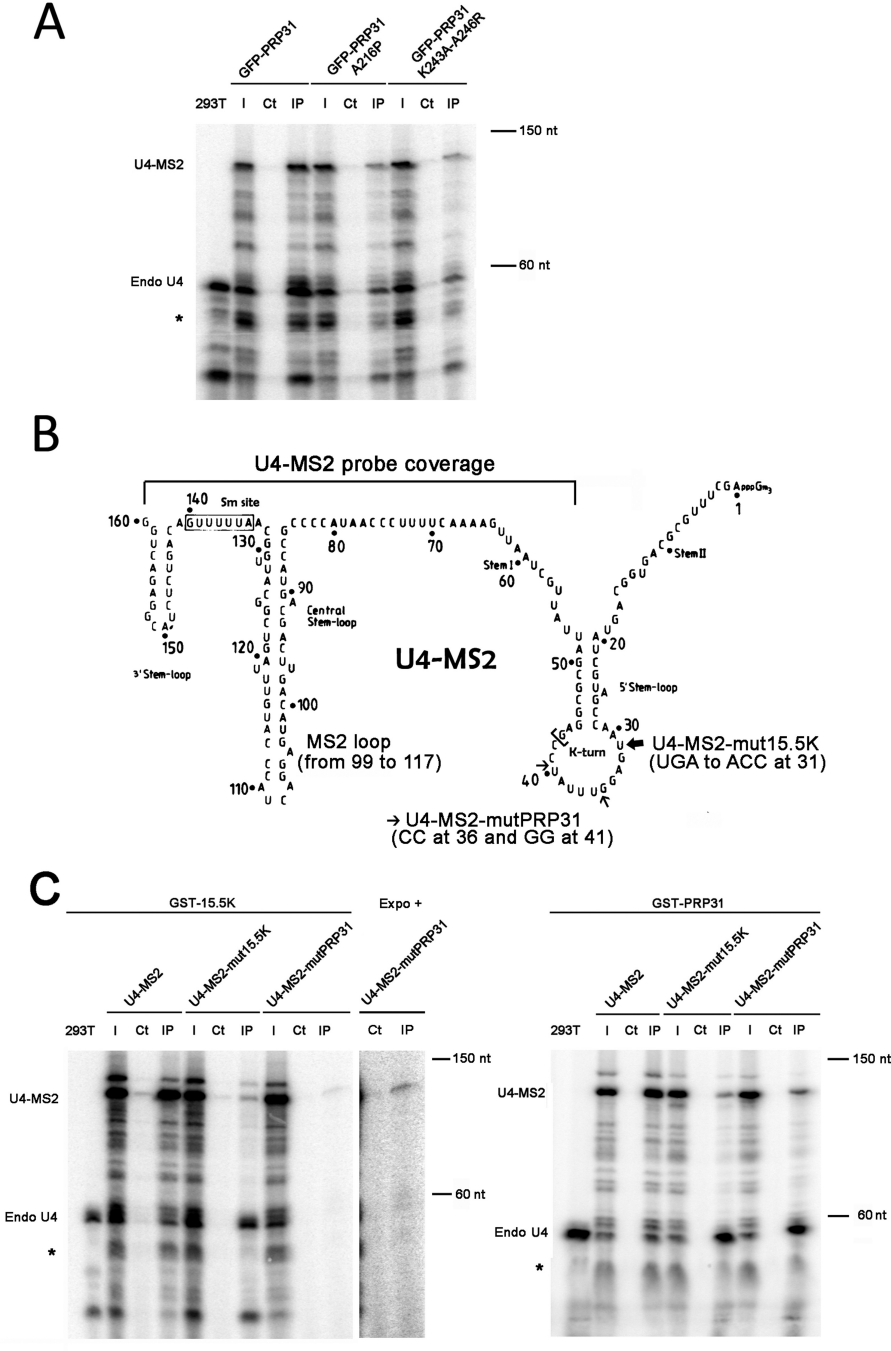
Association of PRP31 and 15.5K with wild-type and mutant U4 snRNAs. **(A)** RNA gel showing the products of an RNase protection assay made with samples from anti-GFP immuno-precipitation of 293T cells, co-transfected with U4-MS2 and GFP-PRP31 (wild-type and indicated mutants). The products corresponding to endogenous U4 and to U4-MS2 are indicated at the left of the gel. Star: additional doublet produced with the U4-MS2 probe. 293T: RNAs from untransfected 293T cells; I: input (5% of pellets); Ct: control immuno-precipitation of the extracts with unconjugated agarose beads; IP: immuno-precipitation of the extracts with GFP-Trap agarose beads. **(B)** Schematic depicting the mutations in U4-MS2 that prevent binding to PRP31 or 15.5K. The MS2 stem-loop (nucleotides 99–117) was inserted in the stem-loop upstream of the Sm site. The mutations affecting binding of PRP31 and 15.5K are indicated and described with small and large arrows, respectively. The region covered by the probe used for the RNase protection assays is indicated (nucleotides 43–160). **(C)** RNA gels showing the products of an RNase protection assay made with samples from GST affinity purification of 293T cells co-transfected with GST-15.5K (left gel) or GST-PRP31 (right gel), and U4-MS2 (wild-type or the indicated mutants). Legends as in (A). An image of higher contrast (expo +) is shown to illustrate the interaction of GST-15.5K with U4-MS2-mutPRP31. 293T: RNAs from untransfected 293T cells; I: input (5% of pellets); Ct: control precipitation of the extracts with unconjugated sepharose 4B beads; IP: GST precipitation of the extracts with sepharose 4B glutathione beads.

Next, we analyzed the interaction of the two mutant PRP31 proteins with U4 snRNA by co-IP experiments (Figure 3A). A modified U4 gene, expressing an RNA with a small tag (U4-MS2; Figure 3B), was transfected into 293T cells together with genes encoding wild-type and mutant GFP-PRP31 proteins. Extracts were then immuno-precipitated with anti-GFP antibodies and analyzed by RNase protection experiments using a probe covering the 3′-end of the modified U4 gene (Figure 3B and Supplemental Figure S1). Endogenous U4 protected two fragments fragment of 56 and 43 nucleotides, and U4-MS2 a fragment of 118 bases (Figure 3A). GFP-PRP31 immuno-precipitated large amounts of both endogenous and tagged U4 RNAs, while the two mutants bound much smaller, but still detectable amounts of these RNAs. This suggested that while the PRP31 mutations prevented stable incorporation of the protein in the U4 RNP, a weak and probably transient association remained. It thus appears that in vivo, PRP31 can interact in two ways with U4: a stable interaction through 15.5K and its canonical binding site, and a novel, weak interaction through an unidentified mechanism.

To further test whether PRP31 could bind U4 without 15.5K, we engineered two mutants of U4 that destroyed binding to either PRP31 alone (U4-MS2-mutPRP31), or to both PRP31 and 15.5K (U4-MS2-mut15.5K; Figure 3B; 32,55). The mutants U4-MS2 snRNAs accumulated in enlarged CBs, consistent with an assembly defect (Supplemental Figure S2; 56). We then directly tested their binding to 15.5K and PRP31 by co-immunoprecipitation. The presence of both endogenous and tagged U4 snRNAs in the transfected cells allowed a direct comparison of the two RNAs. *In vitro*, the binding of PRP31 to U4 RNA requires the presence of 15.5K (55), and none of the mutants were thus expected to bind PRP31 in cells. In the case of wild-type U4-MS2, GST-tagged 15.5K and PRP31 proteins immuno-precipitated this RNA as efficiently as endogenous U4 (Figure 3C; compare the relative intensity of the U4 and U4-MS2 bands in the inputs and pellets). In contrast, GST-PRP31 bound much more efficiently to endogenous U4 than to U4-MS2 mutated in its binding site, while GST-15.5K bound equally well to both RNAs. Finally, U4-MS2 without a binding site for 15.5K interacted much less efficiently than endogenous U4 with either GST-PRP31 or GST-15.5K. However, it is important to note that while this U4 mutant showed decreased binding to GST-PRP31 or GST-15.5K, some weaker binding remained. These observations confirmed that a fraction of PRP31 and 15.5K can associate with U4 snRNA independently of their canonical binding site, and that PRP31 can associate weakly with U4 snRNA without binding 15.5K.

### Wild-type and mutant PRP31 associate with the NUFIP/R2TP chaperone system.

**Figure 4.**
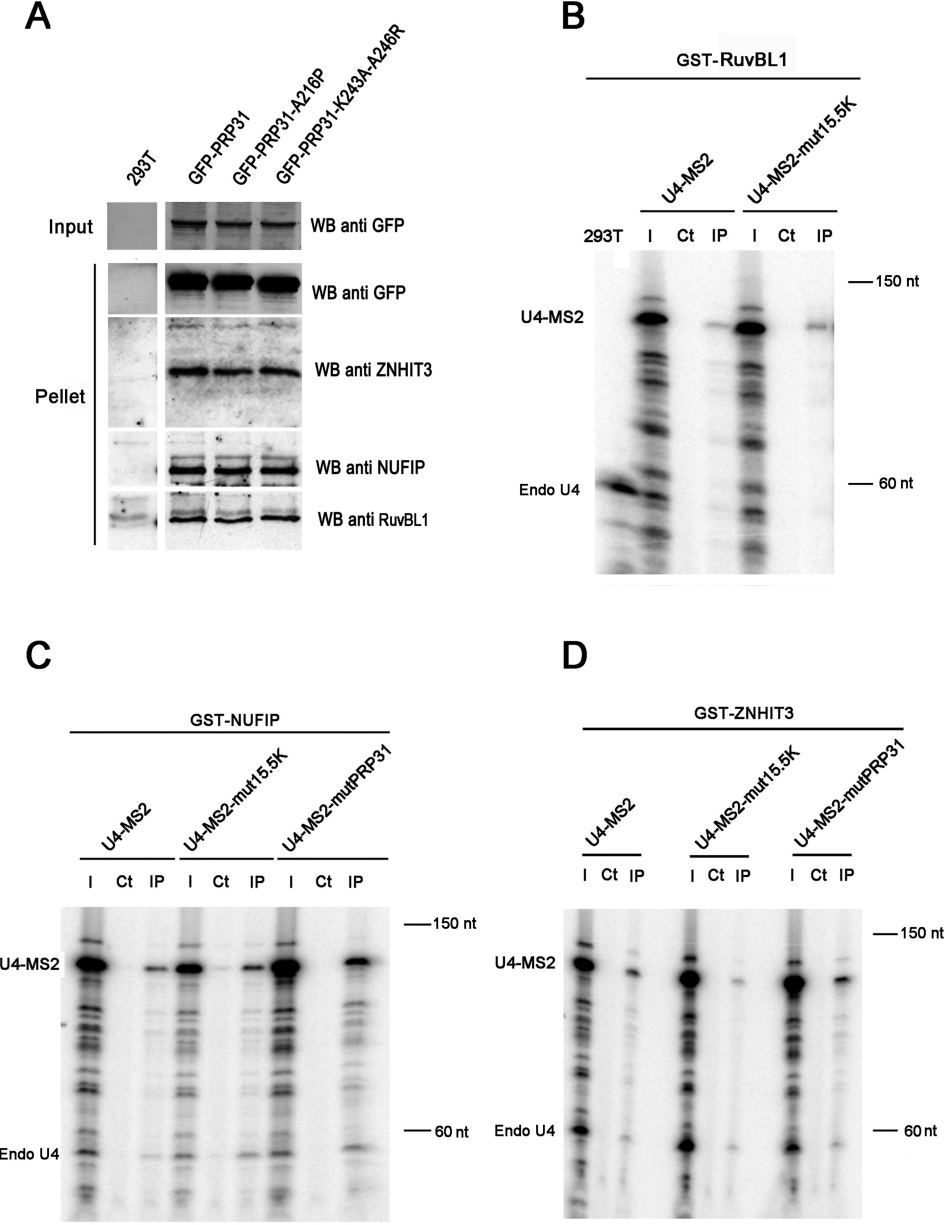
Association of box C/D snoRNP assembly factors with PRP31 and U4 snRNA. **(A)** Western blotting of inputs and pellets of anti-GFP Trap immuno-precipitates of 293T cells, transfected with the indicated GFP-PRP31 fusion proteins. Membrane was cut to separate proteins of different molecular weights and probed with the indicated antibodies. The control corresponding to untransfected 293T cells (293T) was present on the same gel and is shown using a vertical division to remove non-relevant lanes present in the original gel. Input: 5% of pellet. **(B-D)** RNA gels showing the products of an RNase protection assay made with samples from GST precipitations of 293T cells, co-transfected with U4-MS2 (wild-type or indicated mutants), and either GST-RuvBL1 (B), GST-NUFIP (C), or GST-ZNHIT3 (D). The products corresponding to endogenous U4 (Endo U4) and to U4-MS2 are indicated. 293T: RNAs from untransfected 293T cells; I: input (5% of pellets); Ct: control immuno-precipitation of the extracts with sepharose 4B beads; IP: precipitation of the extracts with sepharose 4B glutathione beads.

One possible explanation for the residual binding of mutant PRP31 to wild-type U4, and also for the weak binding of 15.5K and PRP31 to mutant U4 RNAs, was an indirect association of these proteins with U4, which could be mediated by assembly factors. To test this possibility, we first assessed binding of wild-type and mutant PRP31 to ZNHIT3, NUFIP and R2TP proteins (Figure 4A and Supplemental Figure S2B), and then the binding of these assembly factors to wild-type and mutant U4 RNAs (Figure 4C and D). We observed that wild-type and mutant PRP31 associated equally well to NUFIP, ZNHIT3 and RuvBL1. It is interesting to note that the binding of PRP31 A216P to ZNHIT3 was not abolished as observed for the yeast proteins in two-hybrid assays (A226P; Figure 1), but additional indirect interactions occurring in cells could compensate for this mutation. For instance, NUFIP binds both proteins and could bridge them in vivo (this study, 40). Next, we investigated the binding of NUFIP, ZNHIT3 and RuvBL1 to U4. Remarkably, these proteins immuno-precipitated equally well endogenous U4 and the mutant U4-MS2 RNAs (Figure 4B–D). We only observed a small decrease in the binding of ZNHIT3 to U4 mutant that does not bind 15.5K. These data thus raise the possibility that these assembly factors bridge, directly or indirectly, U4 snRNA to PRP31, independently of the 15.5K.

### NUFIP knock-down reduces binding of PRP31 with U4 and leads to its accumulation in Cajal bodies

**Figure 5.**
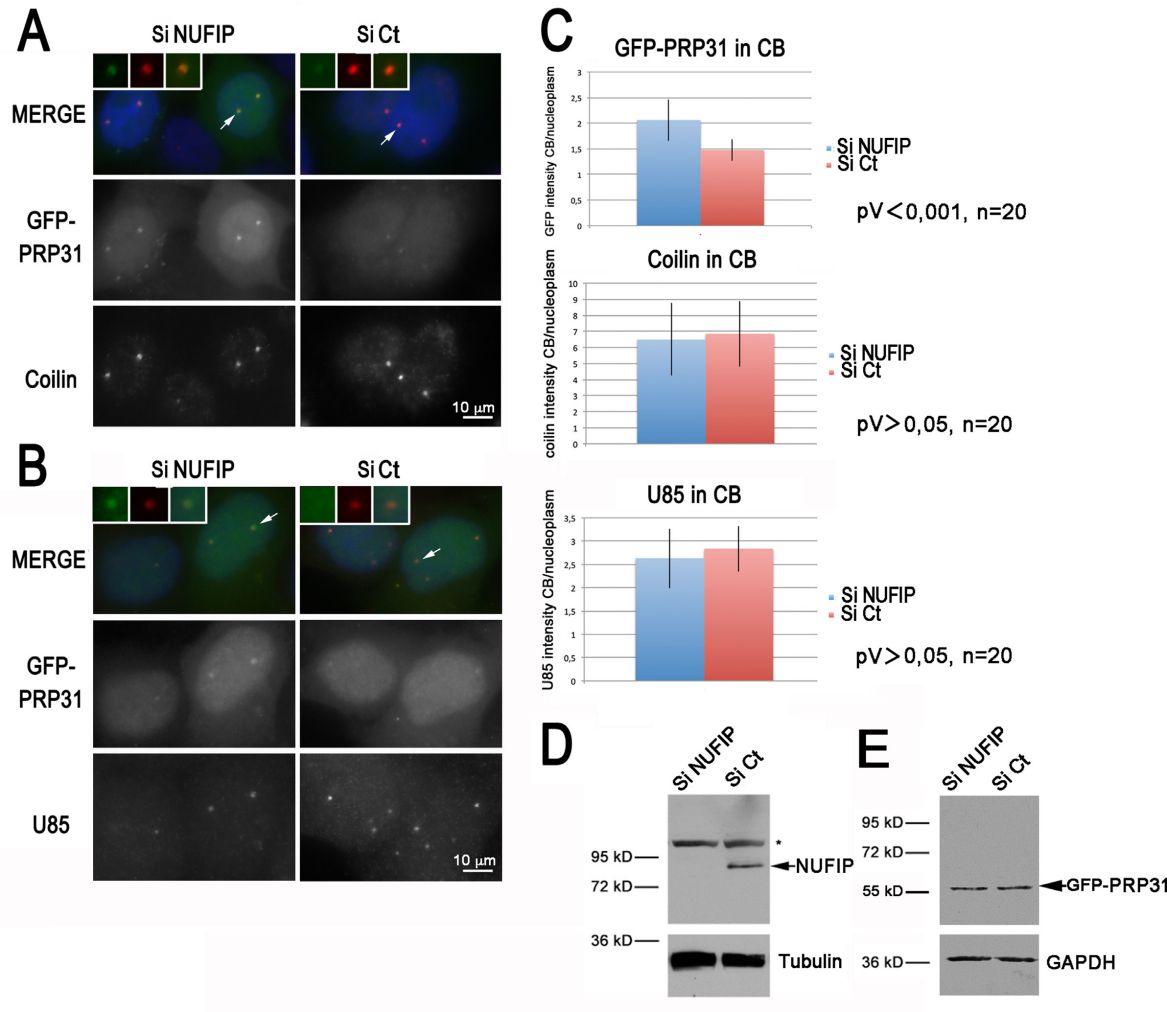
PRP31 accumulates in Cajal bodies following NUFIP depletion. **(A)** Microscopy images of HeLa cells treated with control (Ct) or NUFIP siRNAs. GFP-PRP31 is visible in green, anti-coilin antibody in red, and DAPI in blue. Scale bar is 10 μm. Insets show zoom of selected CBs. **(B)** Microscopy images of HeLa cells treated with control or NUFIP siRNAs. GFP-PRP31 is visible in green, FISH labeling of U85 in red, and DAPI in blue. Scale bar is 10 μm. **(C)** Graph showing the quantification of CB localization of GFP-PRP31, coilin, and U85. Histograms represent an average of signal intensities in CB divided by that of nucleoplasm. **(D)** Western blot of HeLa extracts treated with control and NUFIP siRNAs, and blotted with anti-NUFIP and anti-tubulin antibodies. Star: non-specific band of the NUFIP antibody. **(E)** Same as in (D) but anti-GFP and anti-GAPDH antibodies were used for western blot.

To gain further support to the role of NUFIP in the assembly of U4 snRNP, we generated a stable HeLa cell line expressing GFP-PRP31 and analyzed the localization of the protein following knock-down of NUFIP by siRNAs (Figure 5). In cells treated with a control siRNA, the protein accumulated in the nucleoplasm and was weakly detected in CBs, as previously described for the endogenous protein (23). In cells depleted for NUFIP, the protein was still present in the nucleoplasm, but it accumulated more strongly in CBs. Quantification of the GFP signal intensities showed that the PRP31 protein was 1.5-fold more concentrated in CBs than in the nucleoplasm in control cells, but this ratio raised to 2-fold upon knock-down of NUFIP (*n* = 20, *P*-value < 0,001 with a *t*-test). This effect was specific to PRP31 since in the same cells, the localization of either coilin, the canonical marker of CBs, or U85, a scaRNA that resides in CBs, were not affected (Figure 5A–C). Previous studies showed that interfering with U4/U6:U5 tri-snRNP formation leads to increased levels of PRP31 in CBs (56,57). The accumulation of PRP31 in CBs upon NUFIP knock-down thus suggests that the absence of NUFIP may impair tri-snRNP assembly.

**Figure 6.**
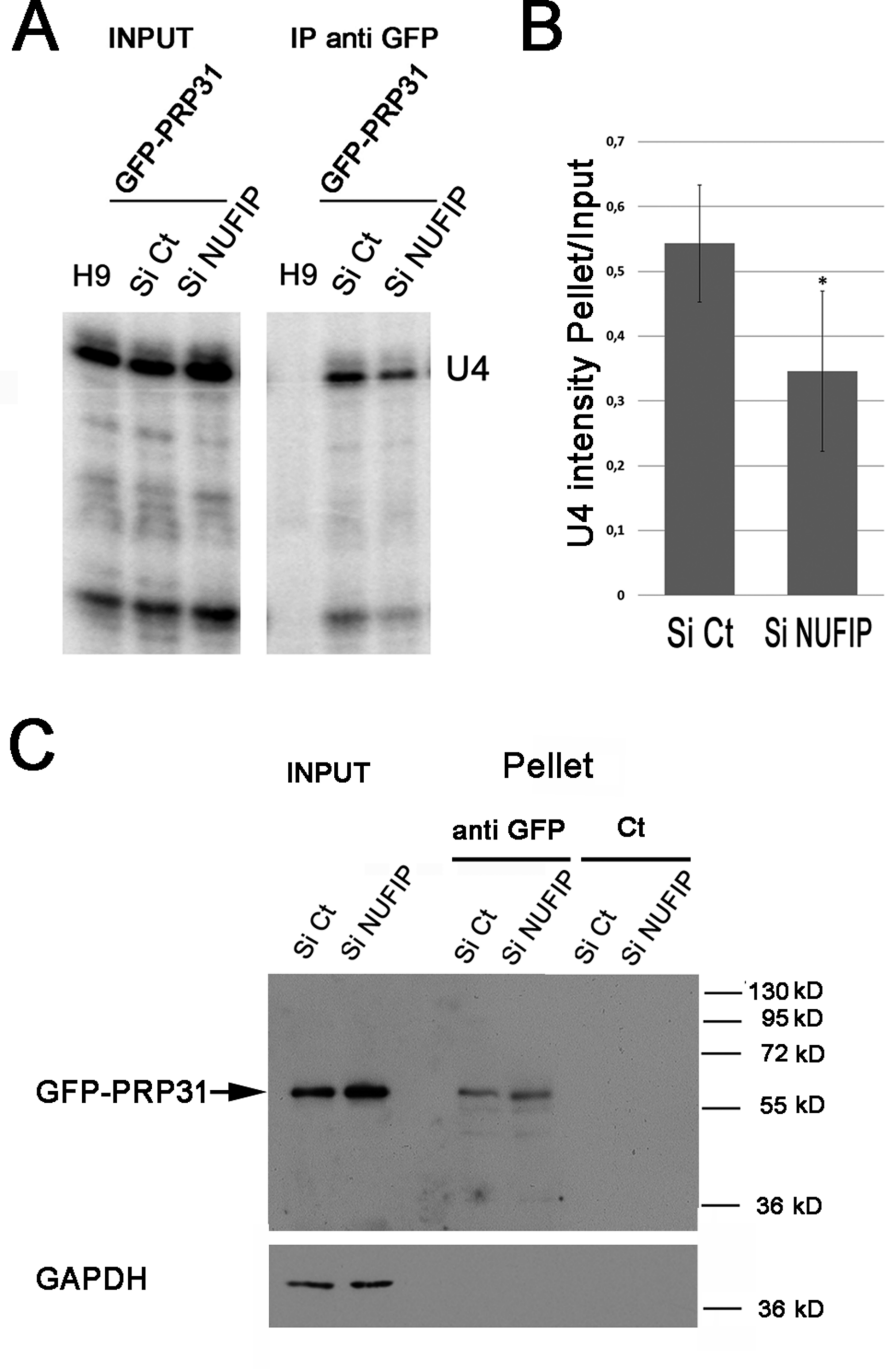
GFP-PRP31 associates less efficiently with U4 snRNA in NUFIP depleted cells. **(A)** RNA gels showing the products of an RNase protection assay made with samples from anti-GFP immuno-precipitations of HeLa cells stably expressing GFP-PRP31, and treated with NUFIP or control (Ct) siRNAs. H9: control cells that do not express GFP-PRP31; IP anti-GFP: pellets after immune-precipitation with agarose GFP-trap beads. **(B)** Graph displaying the quantification of the association of GFP-PRP31 with U4 snRNA. Values are averages of triplicate experiments measuring the ratio of U4 RNA levels in pellets over inputs (± STD). Probability that the two siRNA samples are not different is less than 0.1 (two-sided Student *t*-test). **(C)** Depletion of NUFIP does not affect the efficiency of GFP-PRP31 immuno-precipitation as compared to control. Western blotting of inputs and pellets of anti-GFP immuno-precipitates of cells expressing GFP-PRP31 transfected with siControl or siNUFIP. Western blot was probed with anti-GFP and anti-GAPDH antibodies. Inputs: 5% of pellet.

We then directly measured the association of PRP31 with U4 snRNA (Figure 6). GFP-PRP31 was immuno-precipitated in control and NUFIP knockdown cells, and the amount of U4 snRNA in the input and pellets was measured by RNase protection assays. We show in Figure 6C that depletion of NUFIP did not modify the level of GFP-PRP31 in the cells, nor it modified the amount of immuno-precipitated GFP-PRP31. Nevertheless, association of GFP-PRP31 with U4 snRNA decreased almost 2-fold in the absence of NUFIP (Figure 6A and B), demonstrating a direct role of this factor in the assembly of PRP31 with U4 snRNA.

### SMN associates with wild-type and assembly defective PRP31 proteins

**Figure 7.**
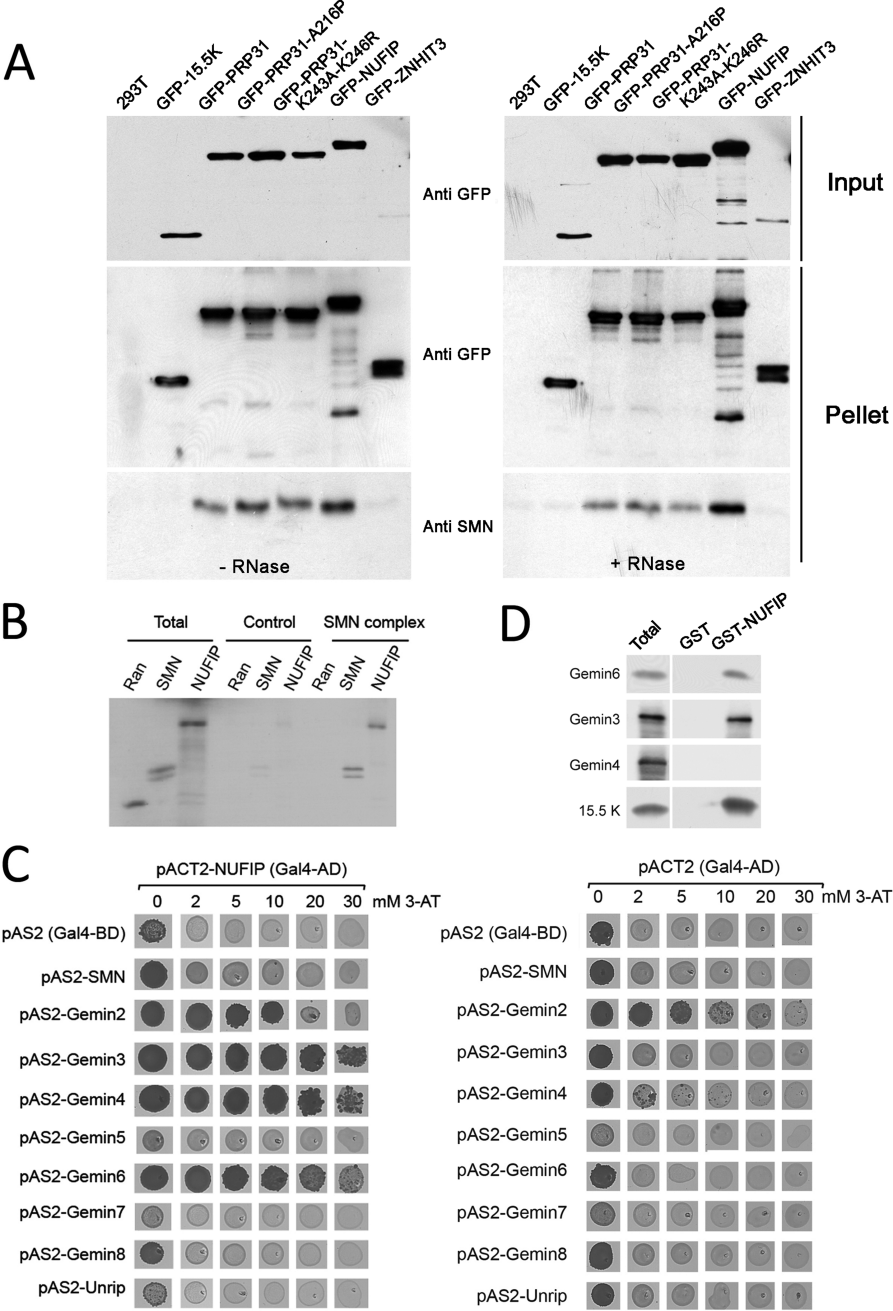
Association of PRP31, NUFIP and ZNHIT3 with the SMN complex. **(A)** Western blotting of inputs and pellets of anti-GFP immuno-precipitates of 293T cells transfected with the indicated GFP constructs. Left gel: no RNase treatment; right gel: RNase treated samples. Membranes are probed with the indicated antibodies. **(B)** Autoradiogram of a gel with input and pellets of immune-precipitates of *in vitro* translated [^35^S]methionine-labeled Ran, SMN and NUFIP (Total; 10%), tested for binding to immobilized SMN complex (SMN complex; 100%). Specific binding was tested with anti-Flag beads pre-incubated with HeLa cell extracts expressing Flag-Gemin2. Non-specific binding was assessed with anti-Flag beads incubated with parental cell extracts that do not express Flag-Gemin2 (Control). **(C)** Micrographs of yeast strains grown in drops on yeast-two hybrid selective media. pACTII-NUFIP was used as prey and the components of the SMN complex used as bait (left panel). Controls were performed with empty pACT2 vector (right panel). Growth on medium lacking histidine indicates an interaction. Increasing amount of 3-Amino-1,2,4-triazol (3-AT), was added to the medium to evaluate the strength of the interaction. **(D)** Western blots of inputs (Total; 10%) and immuno-precipitates (100%) of *E. coli* total extracts expressing recombinant Gemin3, 4, 6 or 15.5K as control, and blotted with the indicated antibodies. Immuno-precipitation was performed in presence of RNase and with beads containing immobilized recombinant GST-NUFIP or GST as control.

Given the general role of the SMN complex in snRNP biogenesis and the role of NUFIP/R2TP in the assembly of U4-specific proteins, we tested possible links between these complexes. First, we assessed interactions by co-immunoprecipitation (Figure 7A). Extracts of 293T cells transfected with GFP-tagged versions of PRP31, 15.5K, NUFIP and ZNHIT3 were purified on GFP-Trap beads, and analyzed by western blotting using anti-SMN antibodies. NUFIP, as well as wild-type and mutant PRP31 proteins, associated with SMN in both RNase treated and untreated extracts (Figure 7A). A weak association was also detected for ZNHIT3, but none for 15.5K (see Supplemental Figure S3A for longer exposure of western blots). These data show that in mammalian cells, the SMN protein associates with PRP31 and NUFIP but not with 15.5K.

### NUFIP associates with the SMN complex through direct interactions with Gemin3 and Gemin6

Next, we investigated whether NUFIP would bind only the SMN protein or the entire SMN complex. We purified the SMN complex from a stable cell line expressing a Flag-tagged version of Gemin2 (46,47; Supplemental Figure S3B), and incubated the purified complex with radiolabeled, *in vitro* translated NUFIP. We found that NUFIP interacted with beads coated with purified SMN complexes, but not with mock-coated beads (Figure 7B). This interaction appeared specific because an unrelated protein, Ran, did not associate with purified SMN complexes.

Next, we attempted to find the proteins responsible for this interaction. We performed systematic yeast-two hybrid tests with NUFIP and components of the SMN complex (Figure 7C). This revealed putative interactions between NUFIP and Gemin3, 4 and 6. To validate these interactions, we performed GST pull-down assays using recombinant proteins. Purified GST or GST-NUFIP immobilized on glutathione beads were mixed with extracts of *E. coli* cells expressing either Gemin3, Gemin4, Gemin6 or 15.5K as positive control. Co-purified proteins were then resolved by gel electrophoresis and analyzed by western blotting (Figure 7D). GST-NUFIP specifically associated with 15.5K, Gemin3 and Gemin6, indicating that the interactions of NUFIP with these proteins are direct.

**Figure 8.**
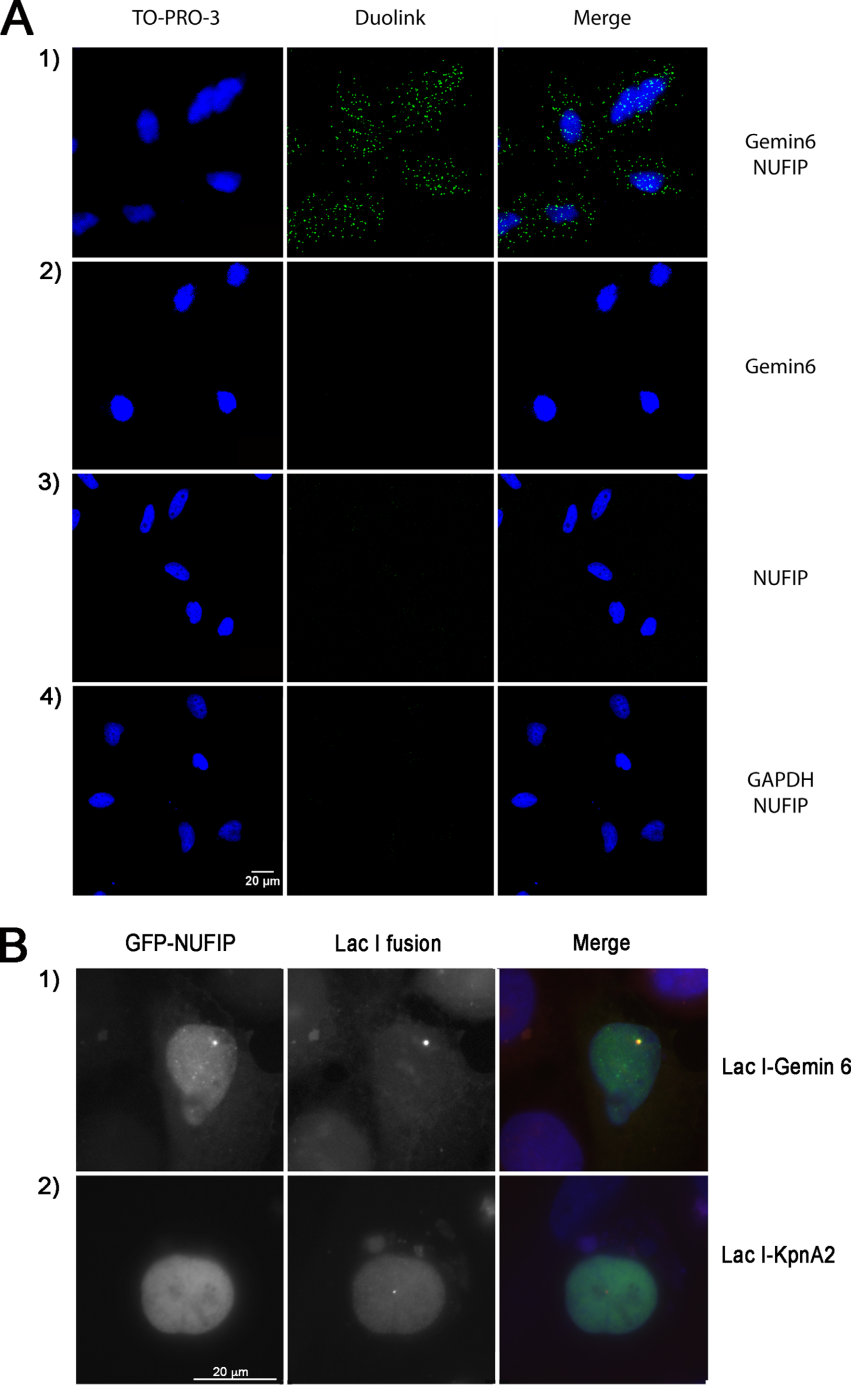
NUFIP associates with Gemin6 in intact HeLa cells. **(A)** Micrographs of HeLa cells fixed and labeled with the PLA (green), using antibodies against the indicated proteins. In controls, one of the primary antibodies was omitted panels on the second and third line. Scale bar, 20 μm. Blue signal demarcate nuclei visualized by TO-PRO-3 staining. **(B)** U2OS LacO cells were co-transfected with GFP-NUFIP and either mRFP–LacI–Gemin6 or mRFP–LacI–KpnA2 used as a negative control. When mRFP–LacI–Gemin6 accumulated at the LacO sites, GFP-NUFIP was also enriched there. In contrast, mRFP–LacI–KpnA2 failed to recruit GFP-NUFIP. Scale bar is 20 μm.

To validate these interactions in a cellular context, we first used PLA (58). This sensitive microscopy assay is based on the use of secondary antibodies coupled to oligonucleotides to create a ligation product, which can form only if the antibodies are close enough to allow for a physical interaction. The ligated products are then amplified using a rolling circle mechanism and detected by fluorescence microscopy. When using specific antibodies directed against NUFIP and Gemin6 (Figure 8A), we detected positive PLA signals in both the cytoplasm and in the nucleus. These were specific because no signals was detected when one of the primary antibody was omitted, or when NUFIP was tested against the abundant GAPDH protein. Positive nuclear and cytoplasmic signals were also detected between NUFIP and other components of the SMN complex (Supplemental Figure S4). This indicated that some NUFIP molecules were located in close vicinity of the entire SMN complex, in both the nucleus and the cytoplasm.

We then focused on interactions within the nucleus and used a spatial co-recruitment assay in U2OS cells, based on the LacI/LacO system (Figure 8B; 48). A cell line containing a tandem array of LacO sites integrated in its genome was transfected with mRFP–LacI–Gemin6 or with mRFP–LacI–KpnA2 expression vectors. When a GFP–NUFIP fusion was co-expressed in these cells, GFP–NUFIP co-localized with the bright spot formed by the binding of mRFP1–LacI–Gemin6 to the LacO sites, but not with the one formed by mRFP1–LacI–KpnA2. This result confirmed that NUFIP and Gemin6 can interact in the nucleus of mammalian cells.

## DISCUSSION

### NUFIP and the HSP90/R2TP chaperone complex mediate assembly of U4-specific proteins

The 15.5K is at the heart of U4 snRNP and C/D snoRNPs. It interacts with PRP31, NOP56 and NOP58, and these three proteins share a very similar structure. Assembly of C/D snoRNPs requires the HSP90/R2TP chaperone system, and HSP90 is required to stabilize NOP58 and 15.5K during this process (39). We show here that NUFIP, a cofactor of the R2TP involved in C/D snoRNP assembly, is also required to assemble PRP31 on U4 snRNA. The 15.5K directly binds NUFIP and NUFIP directly binds the R2TP component PIH1D1 (39,40). Furthermore, PRP31, like NOP58, interacts with NUFIP and with several other components of the HSP90/R2TP system. These data thus suggest a model in which NUFIP, together with the HSP90/R2TP chaperone complex, mediate assembly of the 15.5K and PRP31 proteins on U4 snRNA.

NOP58 and PRP31 play a fundamental structural role in their respective RNP, by making multiple interactions with the RNA and other RNP components (36,37). They appear to also play a central role during the assembly process, as they make multiple interactions with assembly factors (39, this study). NOP58 and PRP31 are modular proteins and through a systematic mutagenesis of yeast Prp31, we show here that these domains appear to mediate partially distinct interactions with assembly factors. Mutants in the Prp31 coiled-coil domain lose interactions with Rvb1, Rvb2 and Rsa1 (the yeast homolog of NUFIP). However, Rvb1 and Rvb2 bind Prp31 mutants affected in the tip domain at the extremity of the coiled-coil, while Rsa1 does not. This suggests that Rvb1/Rvb2 may bind directly to the coiled-coil domain of Prp31, and that Rsa1 may additionally bind the tip domain.

The Rvb1/2 are AAA+ ATPases known to play essential roles in the assembly of box C/D snoRNPs (59). In these RNPs, the coiled-coil domains of NOP56 and NOP58 play a central role in the architecture of the complex by organizing the communication between the two catalytic centers of the snoRNAs (37). Likewise, in the case of U4 snRNP, the coiled-coil domain of PRP31 establishes key interactions essential to the organization of the U4/U6:U5 tri-snRNP (33,36,38). Therefore, our results raise the intriguing possibility that the AAA+ ATPases Rvb1/2 may regulate these interactions. It is also worth noticing that these proteins have been detected in purified fraction of the spliceosome (60). Their roles may thus extend beyond the assembly of U4 snRNP into the formation of the tri-snRNP.

ZNHIT3 and its yeast homolog Hit1 were recently characterized as assembly factors for C/D snoRNPs (40,41). ZNHIT3 makes a stable protein complex with NUFIP and stabilizes it (40,41). Here, we found that ZNHIT3 also associates with PRP31 and that this interaction is conserved in yeast. These data thus suggest that like NUFIP, ZNHIT3 functions in the assembly of both U4 and C/D snoRNPs. Interestingly, we isolated by Y2H a mutant of yeast Prp31p that specifically loses its interaction with Hit1. In human cells, this PRP31 mutant fails to be stably incorporated into U4 snRNP and causes retinitis pigmentosa (50,52,53). This observation suggests a functional role for the ZNHIT3/PRP31 interaction. The function of ZNHIT3 may thus go beyond its role in stabilizing NUFIP (41).

### Assembly of U4-specific proteins may take place in Cajal Bodies

U4 and U6 snRNPs seem to be targeted independently to CBs, where the U4/U6 di-snRNP and the U4/U6-U5 tri-snRNP are preferentially assembled (for review, 30). However, it is currently unknown in which compartment the PRP31 and 15.5K proteins join U4. PRP31 mutants unable to stably associate within U4 snRNP accumulate in the cytoplasm, and this can be explained by two models. First, assembly of PRP31 with U4 snRNA could occur in the cytoplasm, together with the assembly of the Sm-core by the SMN complex. However, PRP31 contains a functional NLS, and the assembly-defective PRP31 mutant A216P is still imported into nuclei at normal rates (54), arguing that its cytoplasmic localization probably results from a re-export of the unassembled protein or to a rapid degradation upon its entry into nuclei. Second, assembly of PRP31 with U4 snRNP could occur in the nucleus. Our PLA assay detects interaction between the SMN complex and NUFIP in both the nucleus and the cytoplasm and thus does not discriminate between the two models. However, U4 mutants that cannot bind the 15.5K accumulate in CBs, thus suggesting that assembly of PRP31 and 15.5K could take place in this compartment as previously proposed for the formation of the di- and tri-snRNP (56 and references therein).

### A possible role for the SMN complex in the assembly of U4-specific proteins

We found that a mutant of PRP31 that is defective for interaction with the 15.5K fails to become stably incorporated in U4, but still associates with NUFIP, RuvBL1 and ZNHIT3. Thus, binding of PRP31 to these factors may precede its stable association with U4 snRNA. In addition, we found that NUFIP, RuvBL1, ZNHIT3 and PRP31 are able to associate with U4 snRNA independently of its binding site for 15.5K, and we also discovered that SMN associates with NUFIP, ZNHIT3 and PRP31. The SMN complex directly binds snRNA through a specific interaction with Gemin5 (61,62). Thus, it could serve as a scaffold to mediate interactions between U4 snRNA, NUFIP, ZNHIT3, and PRP31.

**Figure 9.**
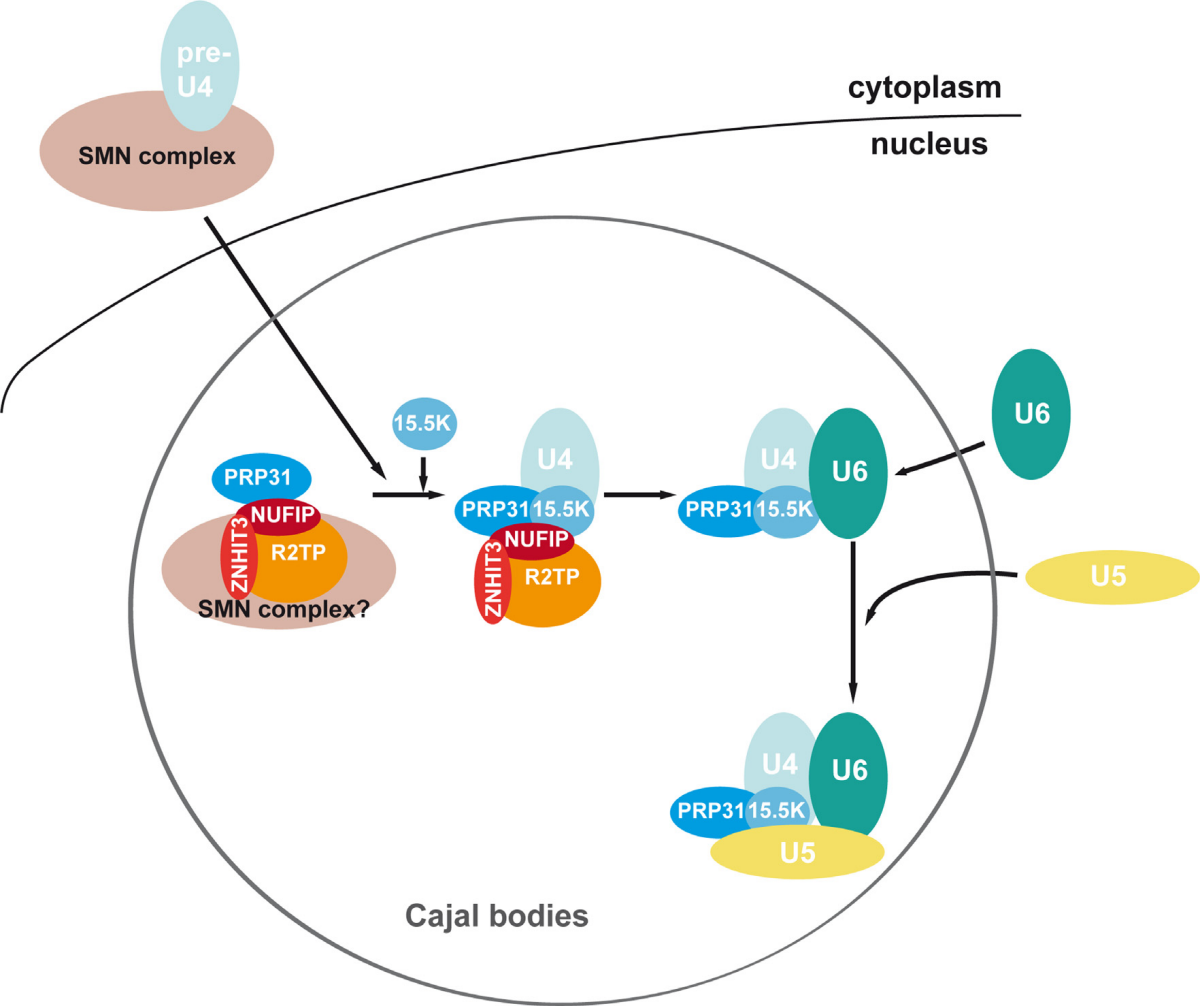
Model for the assembly of U4-specific proteins. The Sm-core containing U4 pre-snRNP is assembled by the SMN complex in the cytoplasm. After its transport into CB, U4 pre-snRNP bind SMN complexes associated with NUFIP, ZNHIT3 and PRP31. Binding of 15.5K then allows stable assembly of PRP31 on U4, and subsequent formation of the di- and tri-snRNP (see the text).

Altogether, the associations detected in this study lead to the hypothesis that the SMN complex may facilitate assembly of U4-specific proteins. We present one model in Figure 9, although alternative possibilities exist. This model has two main components. On one hand, free PRP31 would associate with NUFIP, ZNHIT3 and R2TP, and the resulting complex might also interact with SMN complexes residing in CBs. On the other hand, U4 pre-snRNAs would assemble with the Sm core in the cytoplasm, with the aid of cytoplasmic SMN complexes. Newly assembled U4 RNPs would then be co-imported to the nucleus together with SMN, and transported to CBs (21,56). There, incoming U4 RNPs could interact with CB-associated SMN complex, and this would favor interaction with PRP31, via NUFIP and ZNHIT3. Upon binding of 15.5K, U4 would assemble with PRP31, thereby allowing formation of the di-snRNP and tri-snRNP.

Interestingly, it was shown that the release of SMN from CBs by FGF-2 expression leads to an accumulation of Sm proteins and U4 snRNA in CBs (63). According to our model, this might result from a defect in the assembly of U4-specific proteins. It was also recently shown that U1–70K, a core component of U1 snRNP, associates with the SMN complex in an RNA-independent manner (64). Therefore, the functions of the SMN complex in snRNP biogenesis may extend beyond assembling the Sm core, and may also include assembling snRNP-specific proteins.

The SMN complex has also been proposed to be important for snRNP regeneration following splicing (65). This raises the possibility that some of the interactions reported here between NUFIP, PRP31, and the SMN complex, are involved in the re-assembly of the snRNP-specific proteins, and in particular in the reformation of the di- and tri-snRNP. In line with the present data, it should be pointed out that SMN deficiencies have been proposed to yield a specific defect in the formation of minor and major tri-snRNPs, both in yeasts and vertebrates (66,67). Whether this involves the interactions between NUFIP/R2TP and SMN described here will be an interesting issue to address.

## SUPPLEMENTARY DATA

Supplementary Data are available at NAR online.

SUPPLEMENTARY DATA

## References

[1] Hoskins A., Moore M. (2012). The spliceosome: a flexible, reversible macromolecular machine. Trends Biochem. Sci..

[2] van der Feltz C., Anthony K., Brilot A., Pomeranz Krummel D. (2012). Architecture of the spliceosome. Biochemistry.

[3] Wahl M., Will C., Lührmann R. (2009). The spliceosome: design principles of a dynamic RNP machine. Cell.

[4] Fica S., Tuttle N., Novak T., Li N., Lu J., Koodathingal P., Dai Q., Staley J., Piccirilli J. (2013). RNA catalyses nuclear pre-mRNA splicing. Nature.

[5] Dunn E., Rader S. (2010). Secondary structure of U6 small nuclear RNA: implications for spliceosome assembly. Biochem. Soc. Trans..

[6] Fischer U., Englbrecht C., Chari A. (2011). Biogenesis of spliceosomal small nuclear ribonucleoproteins. Wiley Interdiscip. Rev. RNA.

[7] Battle D., Kasim M., Yong J., Lotti F., Lau C., Mouaikel J., Zhang Z., Han K., Wan L., Dreyfuss G. (2006). The SMN complex: an assembly machine for RNPs. Cold Spring Harb. Symp. Quant. Biol..

[8] Coady T., Lorson C. (2011). SMN in spinal muscular atrophy and snRNP biogenesis. Wiley Interdiscip. Rev. RNA.

[9] Ohno M., Segref A., Bachi A., Wilm M., Mattaj I.W. (2000). PHAX, a mediator of U snRNA nuclear export whose activity is regulated by phosphorylation. Cell.

[10] Hallais M., Pontvianne F., Refsing Andersen P., Clerici M., Lener D., Benbahouche N., Gostan T., Vandermoere F., Robert M., Cusack S. (2013). CBC-ARS2 stimulates 3′-end maturation of multiple RNA families and favors cap-proximal processing. Nat. Struc. Mol. Biol..

[11] Fischer U., Liu Q., Dreyfuss G. (1997). The SMN-SIP1 complex has an essential role in spliceosomal snRNP biogenesis. Cell.

[12] Chari A., Golas M., Klingenhäger M., Neuenkirchen N., Sander B., Englbrecht C., Sickmann A., Stark H., Fischer U. (2008). An assembly chaperone collaborates with the SMN complex to generate spliceosomal SnRNPs. Cell.

[13] Grimm C., Chari A., Pelz J., Kuper J., Kisker C., Diederichs K., Stark H., Schindelin H., Fischer U. (2013). Structural basis of assembly chaperone- mediated snRNP formation. Mol. Cell.

[14] Zhang R., So B., Li P., Yong J., Glisovic T., Wan L., Dreyfuss G. (2011). Structure of a key intermediate of the SMN complex reveals Gemin2's crucial function in snRNP assembly. Cell.

[15] Shpargel K., Matera A. (2005). Gemin proteins are required for efficient assembly of Sm-class ribonucleoproteins. Proc. Natl. Acad. Sci. U.S.A..

[16] Otter S., Grimmler M., Neuenkirchen N., Chari A., Sickmann A., Fischer U. (2007). A comprehensive interaction map of the human survival of motor neuron (SMN) complex. J. Biol. Chem..

[17] Carissimi C., Baccon J., Straccia M., Chiarella P., Maiolica A., Sawyer A., Rappsilber J., Pellizzoni L. (2005). Unrip is a component of SMN complexes active in snRNP assembly. FEBS Lett..

[18] Feng W., Gubitz A., Wan L., Battle D., Dostie J., Golembe T., Dreyfuss G. (2005). Gemins modulate the expression and activity of the SMN complex. Hum. Mol. Genet..

[19] Grimmler M., Otter S., Peter C., Müller F., Chari A., Fischer U. (2005). Unrip, a factor implicated in cap-independent translation, associates with the cytosolic SMN complex and influences its intracellular localization. Hum. Mol. Genet..

[20] Carissimi C., Saieva L., Baccon J., Chiarella P., Maiolica A., Sawyer A., Rappsilber J., Pellizzoni L. (2006). Gemin8 is a novel component of the survival motor neuron complex and functions in small nuclear ribonucleoprotein assembly. J. Biol. Chem..

[21] Narayanan U., Achsel T., Lührmann R., Matera A. (2004). Coupled in vitro import of U snRNPs and SMN, the spinal muscular atrophy protein. Mol. Cell.

[22] Mouaikel J., Verheggen C., Bertrand E., Tazi J., Bordonné R. (2002). Hypermethylation of the cap structure of both yeast snRNAs and snoRNAs requires a conserved methyltransferase that is localized to the nucleolus. Mol. Cell.

[23] Schaffert N., Hossbach M., Heintzmann R., Achsel T., Lührmann R. (2004). RNAi knockdown of hPrp31 leads to an accumulation of U4/U6 di-snRNPs in Cajal bodies. EMBO J..

[24] Darzacq X., Jády B., Verheggen C., Kiss A., Bertrand E., Kiss T. (2002). Cajal body-specific small nuclear RNAs: a novel class of 2′-O-methylation and pseudouridylation guide RNAs. EMBO J..

[25] Klingauf M., Stanek D., Neugebauer K. (2006). Enhancement of U4/U6 small nuclear ribonucleoprotein particle association in Cajal bodies predicted by mathematical modeling. Mol. Biol. Cell.

[26] Staněk D., Rader S., Klingauf M., Neugebauer K. (2003). Targeting of U4/U6 small nuclear RNP assembly factor SART3/p110 to Cajal bodies. J. Cell Biol..

[27] Will C.L., Luhrmann R. (2001). Spliceosomal UsnRNP biogenesis, structure and function. Curr. Opin. Cell Biol..

[28] Achsel T., Brahms H., Kastner B., Bachi A., Wilm M., Luhrmann R. (1999). A doughnut-shaped heteromer of human Sm-like proteins binds to the 3′-end of U6 snRNA, thereby facilitating U4/U6 duplex formation in vitro. EMBO J..

[29] Rader S.D., Guthrie C. (2002). A conserved Lsm-interaction motif in Prp24 required for efficient U4/U6 di-snRNP formation. RNA.

[30] Staněk D., Neugebauer K. (2004). Detection of snRNP assembly intermediates in Cajal bodies by fluorescence resonance energy transfer. J. Cell Biol..

[31] Nottrott S., Hartmuth K., Fabrizio P., Urlaub H., Vidovic I., Ficner R., Lührmann R. (1999). Functional interaction of a novel 15.5kD [U4/U6.U5] tri-snRNP protein with the 5′ stem-loop of U4 snRNA. EMBO J..

[32] Vidovic I., Nottrott S., Hartmuth K., Lührmann R., Ficner R. (2000). Crystal structure of the spliceosomal 15.5kD protein bound to a U4 snRNA fragment. Mol. Cell.

[33] Nottrott S., Urlaub H., Lührmann R. (2002). Hierarchical, clustered protein interactions with U4/U6 snRNA: a biochemical role for U4/U6 proteins. EMBO J..

[34] Watkins N., Ségault V., Charpentier B., Nottrott S., Fabrizio P., Bachi A., Wilm M., Rosbash M., Branlant C., Lührmann R. (2000). A common core RNP structure shared between the small nucleoar box C/D RNPs and the spliceosomal U4 snRNP. Cell.

[35] Mougin A., Gottschalk A., Fabrizio P., Luhrmann R., Branlant C. (2002). Direct probing of RNA structure and RNA-protein interactions in purified HeLa cell's and yeast spliceosomal U4/U6.U5 tri-snRNP particles. J. Mol. Biol..

[36] Liu S., Li P., Dybkov O., Nottrott S., Hartmuth K., Lührmann R., Carlomagno T., Wahl M. (2007). Binding of the human Prp31 Nop domain to a composite RNA-protein platform in U4 snRNP. Science.

[37] Lin J., Lai S., Jia R., Xu A., Zhang L., Lu J., Ye K. (2011). Structural basis for site-specific ribose methylation by box C/D RNA protein complexes. Nature.

[38] Makarova O., Makarov E., Liu S., Vornlocher H., Lührmann R. (2002). Protein 61K, encoded by a gene (PRPF31) linked to autosomal dominant retinitis pigmentosa, is required for U4/U6*U5 tri-snRNP formation and pre-mRNA splicing. EMBO J..

[39] Boulon S., Marmier-Gourrier N., Pradet-Balade B., Wurth L., Verheggen C., Jády B., Rothé B., Pescia C., Robert M., Kiss T. (2008). The Hsp90 chaperone controls the biogenesis of L7Ae RNPs through conserved machinery. J. Cell Biol..

[40] Bizarro J., Charron C., Boulon S., Westman B., Pradet-Balade B., Vandermoere F., Chagot M.E., Hallais M., Ahmad Y., Leonhardt H. (2014). Proteomic and 3D structure analyses highlight the C/D box snoRNP assembly mechanism and its control. J. Cell Biol..

[41] Rothe B., Saliou J.M., Quinternet M., Back R., Tiotiu D., Jacquemin C., Loegler C., Schlotter F., Pena V., Eckert K. (2014). Protein Hit1, a novel box C/D snoRNP assembly factor, controls cellular concentration of the scaffolding protein Rsa1 by direct interaction. Nucleic Acids Res..

[42] Zhao R., Davey M., Hsu Y.C., Kaplanek P., Tong A., Parsons A.B., Krogan N., Cagney G., Mai D., Greenblatt J. (2005). Navigating the chaperone network: an integrative map of physical and genetic interactions mediated by the hsp90 chaperone. Cell.

[43] Bardoni B., Schenck A., Mandel J. (1999). A novel RNA-binding nuclear protein that interacts with the fragile X mental retardation (FMR1) protein. Hum. Mol. Genet..

[44] Charroux B., Pellizzoni L., Perkinson R., Shevchenko A., Mann M., Dreyfuss G. (1999). Gemin3: A novel DEAD box protein that interacts with SMN, the spinal muscular atrophy gene product, and is a component of gems. J. Cell Biol..

[45] Charroux B., Pellizzoni L., Perkinson R., Yong J., Shevchenko A., Mann M., Dreyfuss G. (2000). Gemin4. A novel component of the SMN complex that is found in both gems and nucleoli. J. Cell Biol..

[46] Pellizzoni L., Baccon J., Rappsilber J., Mann M., Dreyfuss G. (2002). Purification of native survival of motor neurons complexes and identification of Gemin6 as a novel component. J. Biol. Chem..

[47] Piazzon N., Rage F., Schlotter F., Moine H., Branlant C., Massenet S. (2008). In vitro and in cellulo evidences for association of the survival of motor neuron complex with the fragile X mental retardation protein. J. Biol. Chem..

[48] Boulon S., Pradet-Balade B., Verheggen C., Molle D., Boireau S., Georgieva M., Azzag K., Robert M., Ahmad Y., Neel H. (2010). HSP90 and its R2TP/Prefoldin-like cochaperone are involved in the cytoplasmic assembly of RNA polymerase II. Mol. Cell.

[49] Richard P., Darzacq X., Bertrand E., Jády B., Verheggen C., Kiss T. (2003). A common sequence motif determines the Cajal body-specific localization of box H/ACA scaRNAs. EMBO J..

[50] Vithana E.N., Abu-Safieh L., Allen M.J., Carey A., Papaioannou M., Chakarova C., Al-Maghtheh M., Ebenezer N.D., Willis C., Moore A.T. (2001). A human homolog of yeast pre-mRNA splicing gene, PRP31, underlies autosomal dominant retinitis pigmentosa on chromosome 19q13.4 (RP11). Mol. Cell.

[51] Utz V.M., Beight C.D., Marino M.J., Hagstrom S.A., Traboulsi E.I. (2013). Autosomal dominant retinitis pigmentosa secondary to pre-mRNA splicing-factor gene PRPF31 (RP11): review of disease mechanism and report of a family with a novel 3-base pair insertion. Ophthalmic Genet..

[52] Deery E.C., Vithana E.N., Newbold R.J., Gallon V.A., Bhattacharya S.S., Warren M.J., Hunt D.M., Wilkie S.E. (2002). Disease mechanism for retinitis pigmentosa (RP11) caused by mutations in the splicing factor gene PRPF31. Hum. Mol. Genet..

[53] Huranová M., Hnilicová J., Fleischer B., Cvacková Z., Stanek D. (2009). A mutation linked to retinitis pigmentosa in HPRP31 causes protein instability and impairs its interactions with spliceosomal snRNPs. Hum. Mol. Genet..

[54] Wilkie S., Morris K., Bhattacharya S., Warren M., Hunt D. (2006). A study of the nuclear trafficking of the splicing factor protein PRPF31 linked to autosomal dominant retinitis pigmentosa (ADRP). Biochim. Biophys. Acta..

[55] Schultz A., Nottrott S., Hartmuth K., Lührmann R. (2006). RNA structural requirements for the association of the spliceosomal hPrp31 protein with the U4 and U4atac small nuclear ribonucleoproteins. J. Biol. Chem..

[56] Novotny I., Malinova A., Stejskalova E., Mateju D., Klimesova K., Roithova A., Sveda M., Knejzlik Z., Stanek D. (2015). SART3-Dependent Accumulation of Incomplete Spliceosomal snRNPs in Cajal Bodies. Cell Rep.

[57] Stanek D., Pridalová-Hnilicová J., Novotný I., Huranová M., Blazíková M., Wen X., Sapra A., Neugebauer K. (2008). Spliceosomal small nuclear ribonucleoprotein particles repeatedly cycle through Cajal bodies. Mol. Biol. Cell..

[58] Söderberg O., Leuchowius K., Gullberg M., Jarvius M., Weibrecht I., Larsson L., Landegren U. (2008). Characterizing proteins and their interactions in cells and tissues using the in situ proximity ligation assay. Methods.

[59] King T.H., Decatur W.A., Bertrand E., Maxwell E.S., Fournier M.J. (2001). A well-connected and conserved nucleoplasmic helicase is required for production of box C/D and H/ACA snoRNAs and localization of snoRNP proteins. Mol. Cell. Biol..

[60] Bessonov S., Anokhina M., Will C.L., Urlaub H., Lührmann R. (2008). Isolation of an active step I spliceosome and composition of its RNP core. Nature.

[61] Battle D., Lau C., Wan L., Deng H., Lotti F., Dreyfuss G. (2006). The Gemin5 protein of the SMN complex identifies snRNAs. Mol. Cell.

[62] Yong J., Kasim M., Bachorik J., Wan L., Dreyfuss G. (2010). Gemin5 delivers snRNA precursors to the SMN complex for snRNP biogenesis. Mol. Cell.

[63] Förthmann B., Brinkmann H., Ratzka A., Stachowiak M., Grothe C., Claus P. (2013). Immobile survival of motoneuron (SMN) protein stored in Cajal bodies can be mobilized by protein interactions. Cell. Mol. Life Sci..

[64] Stejskalova E., Stanek D. (2014). The splicing factor U1-70K interacts with the SMN complex and is required for nuclear gem integrity. J. Cell Sci..

[65] Pellizzoni L., Kataoka N., Charroux B., Dreyfuss G. (1998). A novel function for SMN, the spinal muscular atrophy disease gene product, in pre-mRNA splicing. Cell.

[66] Boulisfane N., Choleza M., Rage F., Neel H., Soret J., Bordonné R. (2011). Impaired minor tri-snRNP assembly generates differential splicing defects of U12-type introns in lymphoblasts derived from a type I SMA patient. Hum. Mol. Genet..

[67] Campion Y., Neel H., Gostan T., Soret J., Bordonné R. (2010). Specific splicing defects in S. pombe carrying a degron allele of the Survival of Motor Neuron gene. EMBO J..

