# Supplementary material for: NUFIP and the HSP90/R2TP chaperone bind the SMN complex and facilitate assembly of U4-specific proteins
Source: Nucleic Acids Res. 2015 Oct 10;43(18):8973–89. doi: 10.1093/nar/gkv809 (PMC4605303; doi:10.1093/nar/gkv809)
Supplement: SUPPLEMENTARY DATA [file supp_43_18_8973__index.html]

NUFIP and the HSP90/R2TP chaperone bind the SMN complex and facilitate assembly of U4-specific proteins — NUFIP and the HSP90/R2TP chaperone bind the SMN complex and facilitate assembly of U4-specific proteins — SUPPLEMENTARY DATA 

# NUFIP and the HSP90/R2TP chaperone bind the SMN complex and facilitate assembly of U4-specific proteins

## SUPPLEMENTARY DATA

- SUPPLEMENTARY DATA
- SUPPLEMENTARY DATA
- SUPPLEMENTARY DATA
- SUPPLEMENTARY DATA
- SUPPLEMENTARY DATA
