# Supplementary material for: NUFIP and the HSP90/R2TP chaperone bind the SMN complex and facilitate assembly of U4-specific proteins
Source: Nucleic Acids Res. 2015 Oct 10;43(18):8973–89. doi: 10.1093/nar/gkv809 (PMC4605303; doi:10.1093/nar/gkv809)
Supplement: SUPPLEMENTARY DATA [file supp_gkv809_nar-00974-y-2015-File012.docx]

**ONLINE SUPPLEMENTAL MATERIAL**

**Supplemental Figure 1.** **RNase protection assays with the U4-MS2 probe.** This probe allows detection of endogeneous U4 by RNase protection assay, proecting fragment of 56 and 43 nucleotides (left gel). Hybridization of the probe with U4-MS2 additionally protects a fragment of 118 nt (right gel). In some experiments, U4-MS2 additionally produces a doublet below 60 nucleotides (marked with stars).

**Supplemental Figure 2. U4 snRNAs mutated in their binding site for PRP31 or 15.5K accumulate in enlarged Cajal bodies**

**(A)** Micrograph of U2OS cells transfected with the indicated U4 construct, and stained with a probe specific for the transfected U4 snRNA (FISH-Cy3), with anti-coilin antibodies (Coilin-FITC), and with DAPI to stain nuclei. Scale bar is 20 μm. **(B)** Western blots of Figure 4A shown entirely. The lanes in the middle are not relevant to this study.

**Supplemental Figure 3. Interaction of SMN with ZNHIT3 and immuno-selection of the SMN complex**

**(A)** Longer exposure of the gel of Figure 7A illustrates interaction of immuno-precipitated GFP-ZNHIT3 with SMN by Western blot. **(B)** Native SMN complexes (SMN complex) were purified by immune-precipitation with anti-Flag antibodies using total extracts prepared from HeLa TET-off cells that stably expressed Flag-tagged Gemin2 as described previously (see Text). The protein composition of the complexes was analyzed by SDS-PAGE followed by silver staining. The non-specific proteins immune-selected from HeLa TET-off cells that do not express Flag-Gemin2 are shown as a negative control (Control). Proteins were identified according to their molecular weight as referred to molecular weight markers (MW). The identity of the proteins was confirmed by Western blotting and mass spectrometry (data not shown).

**Supplemental Figure 4. NUFIP associates with components of the SMN complex in intact HeLa cells.**

Micrographs of HeLa cells fixed and labelled with the Proximity Ligation Assay (PLA; duolink-FITC, green), using antibodies against the indicated proteins. Scale bar is 20 μm. Blue signal are nuclei stained with TO-PRO-3.
