# Supplementary figures and images for: NUFIP and the HSP90/R2TP chaperone bind the SMN complex and facilitate assembly of U4-specific proteins
Source: Nucleic Acids Res. 2015 Oct 10;43(18):8973–89. doi: 10.1093/nar/gkv809 (PMC4605303; doi:10.1093/nar/gkv809)

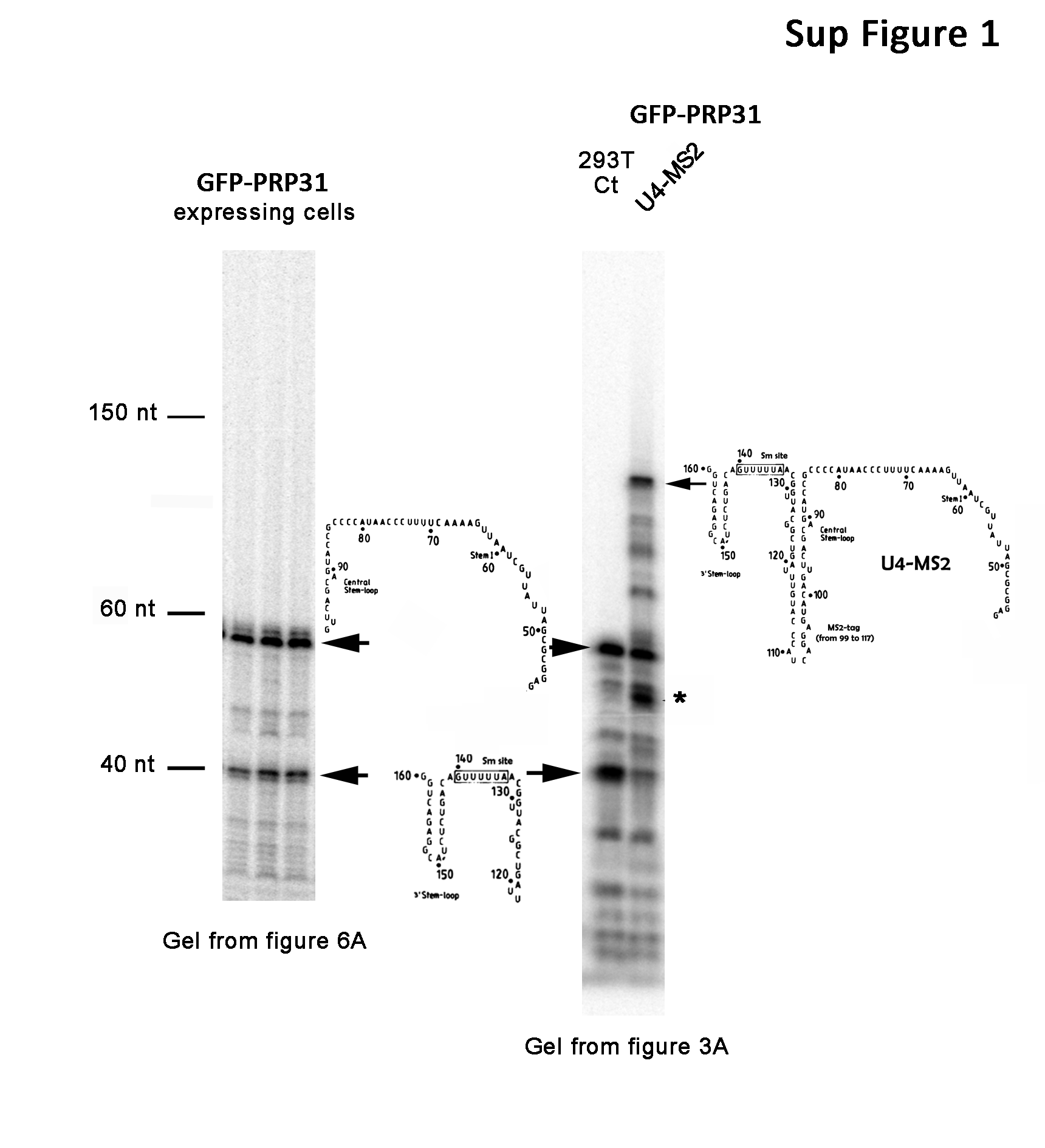

Supplement: SUPPLEMENTARY DATA [file supp_gkv809_nar-00974-y-2015-File013.tif]

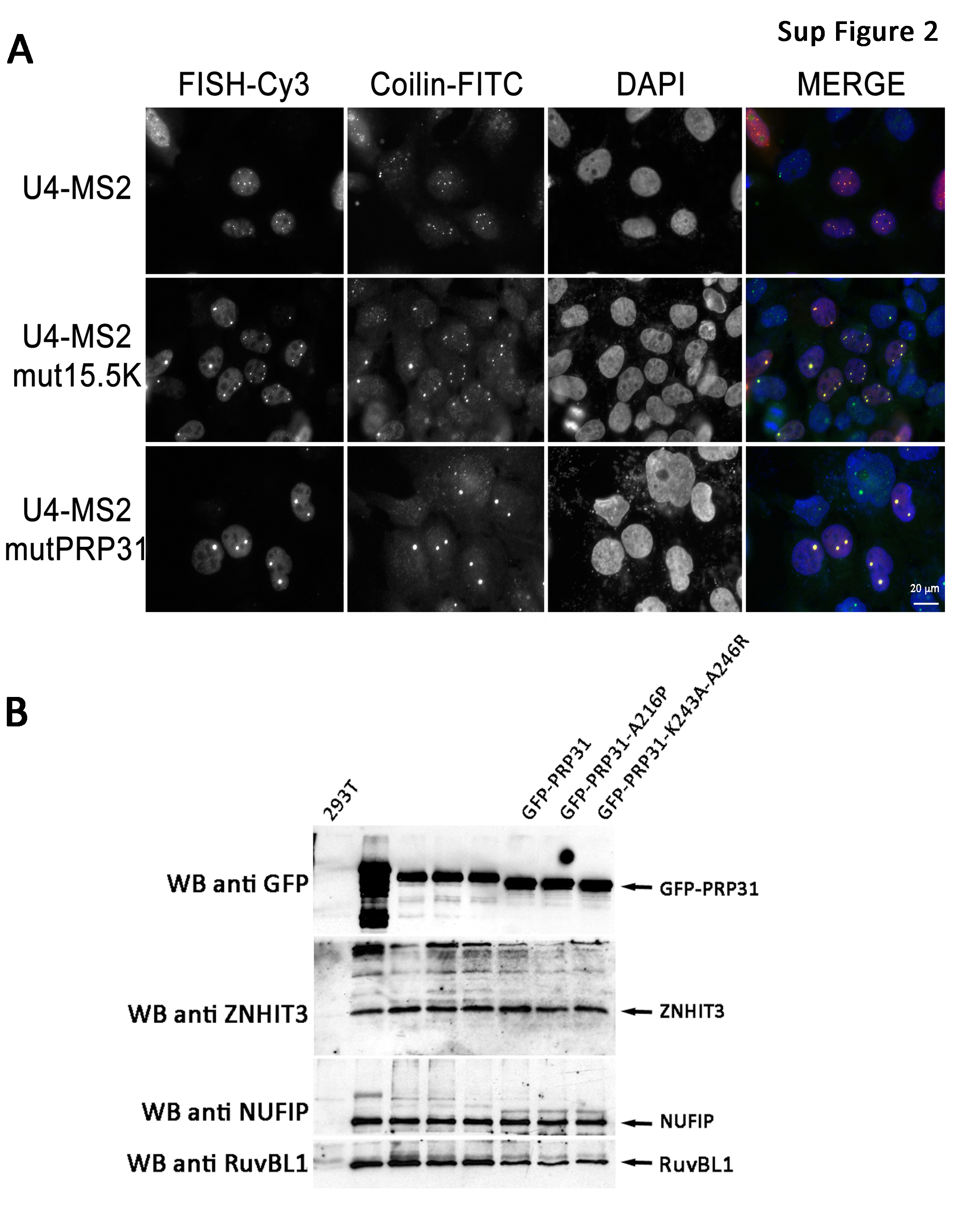

Supplement: SUPPLEMENTARY DATA [file supp_gkv809_nar-00974-y-2015-File014.tif]

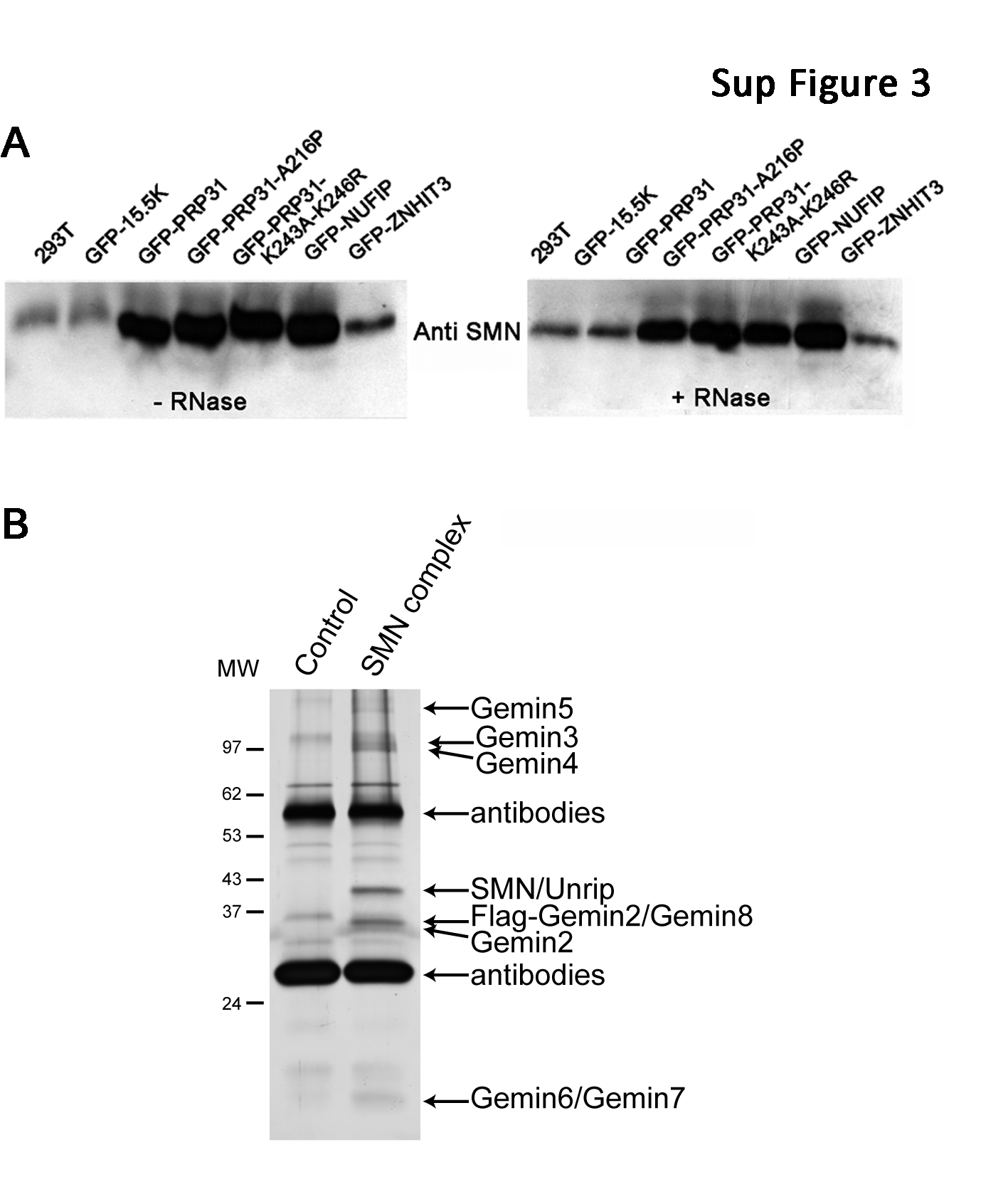

Supplement: SUPPLEMENTARY DATA [file supp_gkv809_nar-00974-y-2015-File015.tif]

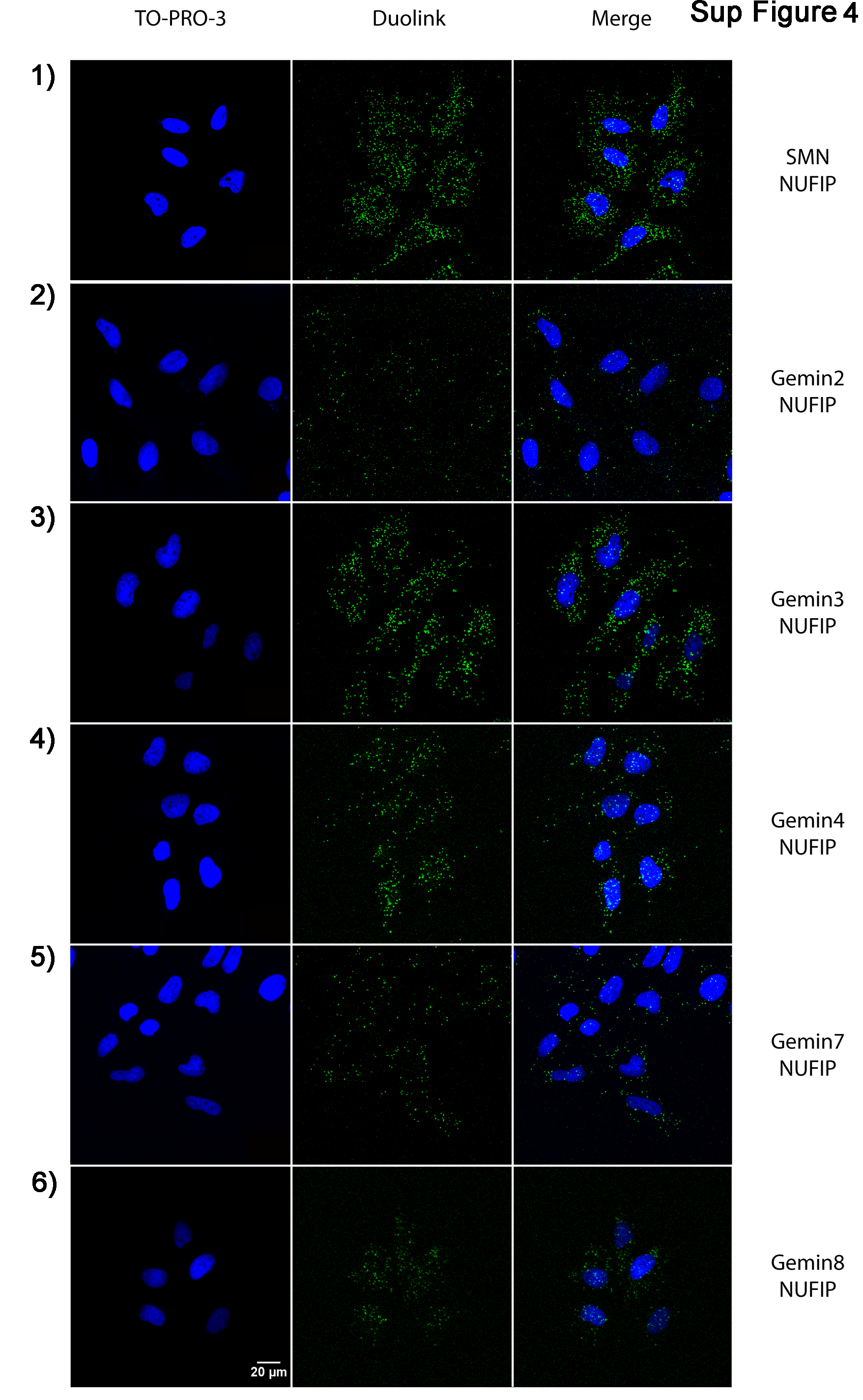

Supplement: SUPPLEMENTARY DATA [file supp_gkv809_nar-00974-y-2015-File016.tif]
